# Impact of plant-based antibiotic alternative supplemented feed on the gut microbiota of Bábolna Tetra-SL chickens experimentally infected with *Salmonella enterica* and *Escherichia coli*

**DOI:** 10.1186/s12917-026-05381-3

**Published:** 2026-02-28

**Authors:** Ádám Kerek, Ábel Szabó, Krisztina Bárdos, Zsófia Bata, Viviána Molnár-Nagy, Ákos Jerzsele, László Ózsvári

**Affiliations:** 1 Department of Pharmacology and Toxicology, University of Veterinary Medicine, Budapest, 1078 Hungary; 2 National Laboratory of Infectious Animal Diseases, Antimicrobial Resistance, Veterinary Public Health and Food Chain Safety, University of Veterinary Medicine, Budapest, 1078 Hungary; 3 Department of Veterinary Forensics and Economics, Institute of Economics and Biostatistics, University of Veterinary Medicine, Budapest, 1078 Hungary; 4 Dr. Bata Zrt, Ócsa, 2364 Hungary

**Keywords:** Phytobiotic feed additive, Fenugreek, Turmeric, Gut microbiota, Chickens, *Salmonella enterica*, *Escherichia coli*, Antibiotic alternatives

## Abstract

**Background:**

*Salmonella enterica* and *Escherichia coli*–associated enteric disturbances contribute to health and productivity losses in poultry and pose zoonotic concerns. In the context of antimicrobial resistance, phytogenic feed additives may offer antibiotic-sparing strategies by modulating the intestinal microbiota. We evaluated a fenugreek (*Trigonella foenum-graecum*) and turmeric (*Curcuma longa*) extract combination for its microbiota-modulating effects in a controlled dual-challenge model.

**Materials and methods:**

A total of 270 Bábolna Tetra-SL chicks were allocated to six groups: low-, medium-, or high-dose phytobiotic; enrofloxacin; infected control; and non-infected control. Birds were orally challenged on days 3–4 post-hatch with clinical isolates of *S. enterica* and *E. coli* (both phenotypically susceptible to enrofloxacin). Cloacal swab samples were collected on days 1, 7, and 42 and profiled by V3-V4 16 S rRNA gene amplicon sequencing. Alpha diversity and beta diversity were assessed in QIIME2 using non-parametric and permutation-based approaches.

**Results:**

Alpha diversity increased with age across groups. On day 42, the medium-dose phytobiotic group exhibited the most balanced community profile among treated groups, whereas enrofloxacin was associated with the strongest early community disruption followed by partial recovery by day 42. Beta diversity ordinations and clustering indicated clear time-driven separation, with treatment-associated differences observed within time points and supported by permutation-based multivariate statistics.

**Conclusions:**

A fenugreek–turmeric phytobiotic modulated cloacal microbiota structure in chickens under a controlled dual-challenge model. Medium-dose supplementation was associated with the most balanced community configuration at the end of the trial, while enrofloxacin induced marked early perturbation. These findings support further evaluation of phytogenic additives as components of antibiotic-reduction strategies in poultry production.

**Supplementary Information:**

The online version contains supplementary material available at 10.1186/s12917-026-05381-3.

## Introduction

*Salmonella enterica* and *Escherichia coli* infections cause substantial economic losses in poultry production worldwide and pose serious public health threats due to their zoonotic potential through the food chain [1]. According to the European Union (EU) zoonoses report for 2022, *Salmonella* remains one of the most frequently reported foodborne pathogens, while avian pathogenic *E. coli* (APEC) infections are among the leading causes of morbidity and mortality in poultry [2].

Decades of widespread antibiotic use for infection control have contributed to the global emergence of antimicrobial resistance (AMR), highlighting the urgent need for sustainable alternatives [3]. In addition to stricter stewardship and preventive measures [4, 5], research into novel compounds is accelerating [6]. Potential strategies include antimicrobial peptides [7], probiotics [8], plant extracts and essential oils [9–13], and other bioactive molecules [14, 15].

Among these, phytobiotics – plant-derived bioactive compounds – have gained increasing attention as promising feed additives that can improve performance, modulate the gut microbiota, and reduce pathogen colonization [16]. Several herbs, including oregano, rosemary, garlic, and fenugreek, have demonstrated in vivo efficacy in mitigating *Salmonella* and *E. coli* colonization, thereby lowering zoonotic risk [17, 18]. Nevertheless, their widespread adoption is limited by challenges such as optimal dosing, extraction methods, and application strategies, which remain poorly standardized [19].

Fenugreek (*Trigonella foenum-graecum*) seeds are rich in saponins, flavonoids, and mucilage with immunomodulatory, antimicrobial, and prebiotic properties. Supplementation has been shown to increase beneficial genera such as *Lactobacillus* and *Bacteroides* while reducing potential pathogens, supporting gut microbial balance [20–22]. Experimental studies further suggest protective effects against *E. coli* infection [23] and improvements in growth, feed conversion [24], and cecal microbiota composition [25]. Turmeric (*Curcuma longa*), widely used in traditional Asian medicine and cuisine, has attracted increasing scientific attention due to its multifunctional bioactive compound, curcumin. Curcumin is a hydrophobic polyphenol with well-documented antioxidant, anti-inflammatory, and anti-carcinogenic activities demonstrated in both in vitro and in vivo models [26–29]. Beyond these pleiotropic effects, curcumin has been consistently shown to protect and stabilize the intestinal mucosal barrier in a range of gastrointestinal disease models in humans and animals [30–32]. These properties, together with its favorable safety profile, position curcumin (and turmeric-derived curcuminoids) as a particularly promising candidate for incorporation into phytobiotic feed additives [26, 33].

The gut microbiota plays a critical role in nutrient digestion, immune development [34], and colonization resistance [35]. Advances in high-throughput sequencing, particularly 16 S rRNA amplicon sequencing, have enabled detailed taxonomic profiling and interaction analyses within microbial communities [36]. While fenugreek has been studied in the context of poultry growth and immunity [37], little is known about its effects on the gut microbiota under co-infection with *S. enterica* and *E. coli*. Recent work by Kerek et al. [38] showed that low-dose supplementation of the phytobiotic additive reduced *Salmonella* shedding and improved gut morphology, but a deeper understanding of microbial community shifts requires next-generation sequencing [38].

Here, we investigated how phytobiotic-supplemented feed modulates gut microbiota composition in Bábolna Tetra-SL chickens subjected to a controlled Gram-negative challenge with *S. enterica* and a clinical *E. coli* isolate, using 16 S rRNA gene amplicon sequencing. The inclusion of the *E. coli* isolate was intended to increase enterobacterial pressure in a field-relevant co-challenge setting and to assess microbiota resilience under polymicrobial exposure, rather than to imply that *E. coli* per se is uniformly pathogenic. We hypothesized that phytobiotic supplementation would promote a microbiota profile consistent with improved community stability, operationally defined as higher diversity/evenness and reduced Enterobacterales dominance together with an increased relative abundance of common commensal taxa typically associated with gut homeostasis (e.g., *Lactobacillus* spp.). In intensive poultry production, *Salmonella* exposure frequently co-occurs with increased intestinal Enterobacterales load and opportunistic overgrowth of *E. coli*, contributing to dysbiosis and impaired colonization resistance. Therefore, we applied a controlled dual-challenge model using *S. enterica* and a clinical *E. coli* isolate to mimic a field-relevant Gram-negative pressure and to evaluate whether phytobiotic supplementation supports microbiota resilience compared with antibiotic treatment.

## Materials and methods

### Animals and housing

A total of 270 Bábolna Tetra-SL hybrid day-old chicks (Bábolna Tetra Kft., Szarka Ferenc Hatchery, Hungary) were purchased for the study, with a 1:1 sex ratio. All procedures were approved by the Institutional Animal Care and Use Committee of the University of Veterinary Medicine, Budapest, Hungary (license number: PE/EA/01174-6/2023), and complied with national animal welfare regulations. At hatch, chicks received standard vaccinations against Marek’s disease, Newcastle disease, and infectious bronchitis. Birds were housed under controlled environmental conditions in the accredited animal facility of the Department of Pharmacology and Toxicology at the University of Veterinary Medicine Budapest, and were fed a standard basal diet. The phytobiotic additive (Dr. Bata Ltd., Ócsa, Hungary) is a proprietary formulation containing *Trigonella foenum-graecum* (fenugreek) and *Curcuma longa* (turmeric) extracts on a carrier base. Due to commercial confidentiality, the manufacturer does not disclose the exact extract ratios and does not provide standardization data for specific marker compounds (e.g., total saponins/curcuminoids). However, the guaranteed analysis listed on the product label (publicly available) was provided by the manufacturer and is routinely verified as part of internal quality control: moisture ≤ 8.0%, crude protein ≥ 18.0%, crude fiber ≥ 15.0%, crude ash ≥ 10.0%, and copper (Cu) ≥ 3,500 mg/kg. The additive was incorporated into the basal diet at the designated inclusion rates by the feed manufacturer (Dr. Bata Ltd., Hungary) (Supplementary Tables S1–S2).

### Experimental design and group

Chicks were randomly allocated to 18 experimental groups (15 birds/group) and housed in separate pens in physically separated rooms/compartments within the animal facility of the Department of Pharmacology and Toxicology, University of Veterinary Medicine Budapest. Biosecurity measures (dedicated equipment, personnel protective clothing, and disinfection protocols) were applied to prevent cross-contamination between groups. The experimental design included six treatment groups with three replicates each: negative control (uninfected, untreated), positive control (infected, untreated), enrofloxacin-treated (10 mg/L in drinking water for five days post-infection, corresponding to an approximate daily dose of 10–15 mg/kg body weight, based on standard water intake of broiler chickens; Tolnagro Kft., Szekszárd, Hungary), and phytobiotic-supplemented groups receiving low (0.1 g/kg), medium (1 g/kg), or high (10 g/kg) doses of the newly developed feed additive from day 1 (i.e., starting before experimental infection) and continued throughout the study (Supplementary Table S3).

All infection-related procedures were conducted under controlled-access animal facility conditions with enhanced biosecurity measures (PPE, disinfection, dedicated equipment, and waste handling) appropriate for handling enteric bacterial challenge strains.

### Infection protocol

*S. enterica* subsp. *enterica* serovar Enteritidis and *E. coli* challenge strains used for experimental infection were clinical isolates provided by the National Food Chain Safety Office – Animal Health and Diagnostic Directorate (Nébih-ÁDI), Hungary, originating from poultry-related diagnostic submissions. Internal strain identification numbers were assigned by the diagnostic laboratory; however, no public reference strain numbers were available.

The *E. coli* isolate was not characterized as a defined pathogenic pathotype prior to the experiment. Its inclusion was intentional and aimed at increasing enterobacterial pressure in a field-relevant co-challenge model, rather than inducing overt colibacillosis. This approach reflects conditions commonly encountered in poultry production, where commensal or opportunistic *E. coli* populations contribute to dysbiosis and interact with other enteric pathogens.

Antimicrobial susceptibility testing was conducted exclusively for enrofloxacin using broth microdilution, given its relevance in poultry practice; both isolates were confirmed to be phenotypically susceptible. No additional antimicrobial resistance profiling was undertaken.

Strains were first cultured on Tryptic Soy Agar (Biolab Zrt., Budapest, Hungary) and subsequently grown in Tryptic Soy Broth (TSB) at 37 °C for 4 h to reach a final concentration of approximately 10^8^ CFU/mL. On days 3 and 4 post-hatch, each bird received 0.5 mL of the bacterial suspension in TSB by oral gavage, corresponding to an inoculum of approximately 5 × 10^7^ CFU per bird per administration for *Salmonella* and for *E. coli*, respectively. In the antibiotic group, enrofloxacin treatment was initiated the day after the second infection and continued for five consecutive days.

### Bacteriological confirmation of *Salmonella *colonization

To verify successful *S. enterica* challenge, bacteriological re-isolation was performed from cloacal swab samples. Swabs were pre-enriched in buffered peptone water (37 °C, 18–24 h), followed by selective enrichment and plating on *Salmonella*-selective agar. Presumptive colonies were confirmed using conventional biochemical identification according to routine diagnostic procedures. This approach was used as qualitative confirmation of colonization, recognizing that 16 S rRNA short-amplicon sequencing may lack sensitivity for low-abundance *Salmonella*.

Selective plating was performed on Salmonella-selective agar media (Rambach agar, Chebio Kft., Budapest, Hungary), and plates were incubated at 37 °C for 24 h. Presumptive *Salmonella* colonies were identified based on characteristic colony morphology, followed by confirmation using conventional biochemical identification methods in accordance with routine diagnostic protocols.

Bacteriological re-isolation was applied as a qualitative confirmation of intestinal colonization and was not intended for quantitative enumeration or comparative assessment between experimental groups.

### Sample collection and DNA extraction

Samples were collected at three time points (days 1, 7, and 42) using sterile cloacal swabs obtained from live birds (i.e., excreted droppings were not used and no necropsy sampling was performed for microbiota profiling). Immediately after collection, each swab was rinsed into a sterile microcentrifuge tube (Biolab Zrt., Budapest, Hungary) containing sterile phosphate-buffered saline (PBS), and samples were frozen without delay and stored at − 80 °C until DNA extraction; thus, samples were not kept at room temperature for prolonged periods prior to processing.

Birds were initially allocated at 15 per group; due to early mortality, the final number of birds available for longitudinal sampling was 12 per group. At each time point, six birds per group were randomly selected and pooled into two composite samples (three swabs per composite), yielding a total of 108 composite samples (18 groups × 3 time points × 2 composites).

DNA was extracted using the IndiSpin Pathogen Kit (Indical Bioscience, Germany). DNA yield was quantified using a Take3 plate on a BioTek Synergy H1 Multimode Reader (Agilent, USA), and DNA extracts were stored at − 20 °C until library preparation and sequencing.

Early mortality occurred during the study; all deceased birds underwent necropsy and gross lesions were recorded, with a group-level summary provided in Supplementary Table S4.

### Library preparation, sequencing

The V3–V4 region of the bacterial 16 S rRNA gene was amplified using primers 341 F (CCTAYGGGRBGCASCAG) and 806R (GGACTACNNGGGTATCTAAT) with sample-specific barcodes. PCR reactions were performed in 15 µL volumes using Phusion High-Fidelity PCR Master Mix (Thermo Fisher Scientific, USA), with 10 ng template DNA and 0.2 µM of each primer. Thermal cycling consisted of an initial denaturation at 98 °C for 1 min, followed by 30 cycles of 98 °C for 10 s, 50 °C for 30 s, and 72 °C for 30 s, with a final extension at 72 °C for 5 min. Amplicons were purified using magnetic beads (AMPure XP; Beckman Coulter, USA), quantified using a fluorometric method (Qubit dsDNA HS Assay; Thermo Fisher Scientific, USA), and pooled in equimolar amounts based on amplicon concentration. Libraries were subjected to paired-end sequencing (2 × 250 bp Illumina chemistry) at Novogene (Tianjin, China). A no-template PCR control was not included and sequenced in the present study [39].

### Bioinformatics

Raw reads were processed following a standard QIIME2 (v2022.2) pipeline. Low-quality reads were trimmed with fastp (v0.23.1) [40] and paired-end reads were merged with FLASH (v1.2.11) [39]. Chimeric sequences were removed using vsearch (v2.16.0) [41]. Noise reduction and amplicon sequence variant (ASV) inference were performed with the DADA2 plugin [42]. Taxonomic classification was conducted using the classify-sklearn Naive Bayes algorithm in QIIME2 against the SILVA 138.1 SSURef NR99 database [40, 43], supplemented with NCBI taxonomy when needed [44].

### Diversity analysis

Alpha diversity was assessed in QIIME2 using a set of complementary indices selected to capture distinct ecological properties of the microbial communities. Observed features (amplicon sequence variants, ASVs) and Chao1 were used to estimate richness, Shannon and Simpson indices to account for both richness and evenness, Pielou’s evenness to specifically describe community evenness, and Dominance to identify potential overrepresentation of individual taxa. Good’s coverage was calculated to evaluate sampling completeness. Rarefaction and species accumulation curves were applied to assess sequencing depth and sampling adequacy [45].

Beta diversity was assessed using weighted and unweighted UniFrac distance metrics [46, 47]. Visualization was performed with heatmaps, UPGMA clustering, PCA, PCoA, and NMDS, implemented in R (packages *vegan*, *ade4*, *ggplot2*). These clustering and ordination visualizations are provided as complementary, exploratory summaries; formal statistical support for between-group differences is based on PERMANOVA/ANOSIM/MRPP results reported in the Supplementary Tables.

### Statistical analysis of community differences

Group-level differences in community structure were tested using ANOSIM, permutational MANOVA (Adonis), and MRPP [48, 49]. Taxon contributions to between-group dissimilarities were quantified using SIMPER [50].

To ensure a pre-specified and coherent analytical workflow, the primary outcomes were alpha diversity indices and overall community structure (beta diversity) assessed at each sampling time point. Primary comparisons were performed between the infected untreated control and each intervention group at days 7 and 42, while day 1 ( (pre-challenge) served as a baseline reference. Community-level differences were tested using PERMANOVA on UniFrac distance matrices, and dispersion was evaluated to exclude heterogeneity-driven artefacts. Secondary analyses included differential abundance testing at genus level with multiple-testing correction (FDR). All analyses were conducted using a consistent pipeline, and only methods with corresponding results are retained in the manuscript. For pairwise comparisons, *p*-values were adjusted for multiple testing using the Benjamini–Hochberg false discovery rate (BH–FDR) procedure.

## Results

### Confirmation of *Salmonella* colonization

Successful *S. enterica* colonization was confirmed by bacteriological re-isolation from cloacal swab samples collected after experimental challenge. Following selective enrichment and plating, colonies consistent with *Salmonella* spp. were recovered and confirmed by biochemical identification. These findings verify effective intestinal exposure and colonization in challenged birds, supporting the validity of the infection model applied for subsequent microbiota analyses.

### Clinical observations and mortality

Birds were monitored daily throughout the experiment for general health status. No systematic clinical scoring or production performance endpoint (e.g., body weight gain) was collected as part of the microbiota-focused study design. Early mortality occurred during the study; all deceased birds underwent necropsy and gross lesions were recorded, with a group-level summary provided in Supplementary Table S4. Following adjustment for early mortality, 12 birds per group remained available for longitudinal sampling.

### Sequencing quality

The 16 S rRNA amplicon sequencing generated an average of 0.104 million paired-end raw reads per sample. After merging, 0.103 million reads remained, of which 0.101 million passed quality filtering. Removal of chimeras resulted in an average of 0.092 million high-quality reads per sample available for downstream analyses. DADA2 denoising yielded a total of 5,185 unique amplicon sequence variants (ASVs) across the dataset. The mean read length was 428 bp, with an average GC content of 53.8%. Data quality was high, with Q20 and Q30 scores of 98.6% and 95.1%, respectively, ensuring reliability for bioinformatic processing (Supplementary Table S5).

Species accumulation curves did not reach a clear plateau, indicating that additional sampling could reveal further low-abundance taxa (Supplementary Fig. S1). Nevertheless, sequencing quality and downstream filtering yielded robust community profiles for comparative analyses across groups processed under identical conditions.

### Alpha diversity

Alpha diversity indices were calculated in QIIME2 and compared across treatment groups at each time point using the Kruskal–Wallis test followed by Dunn’s post hoc test with false discovery rate (FDR) correction (Supplementary Table S6-S7). A total of 108 composite samples were planned; 107 produced sufficient sequencing output and were retained for downstream analyses.

**Fig. 1:**
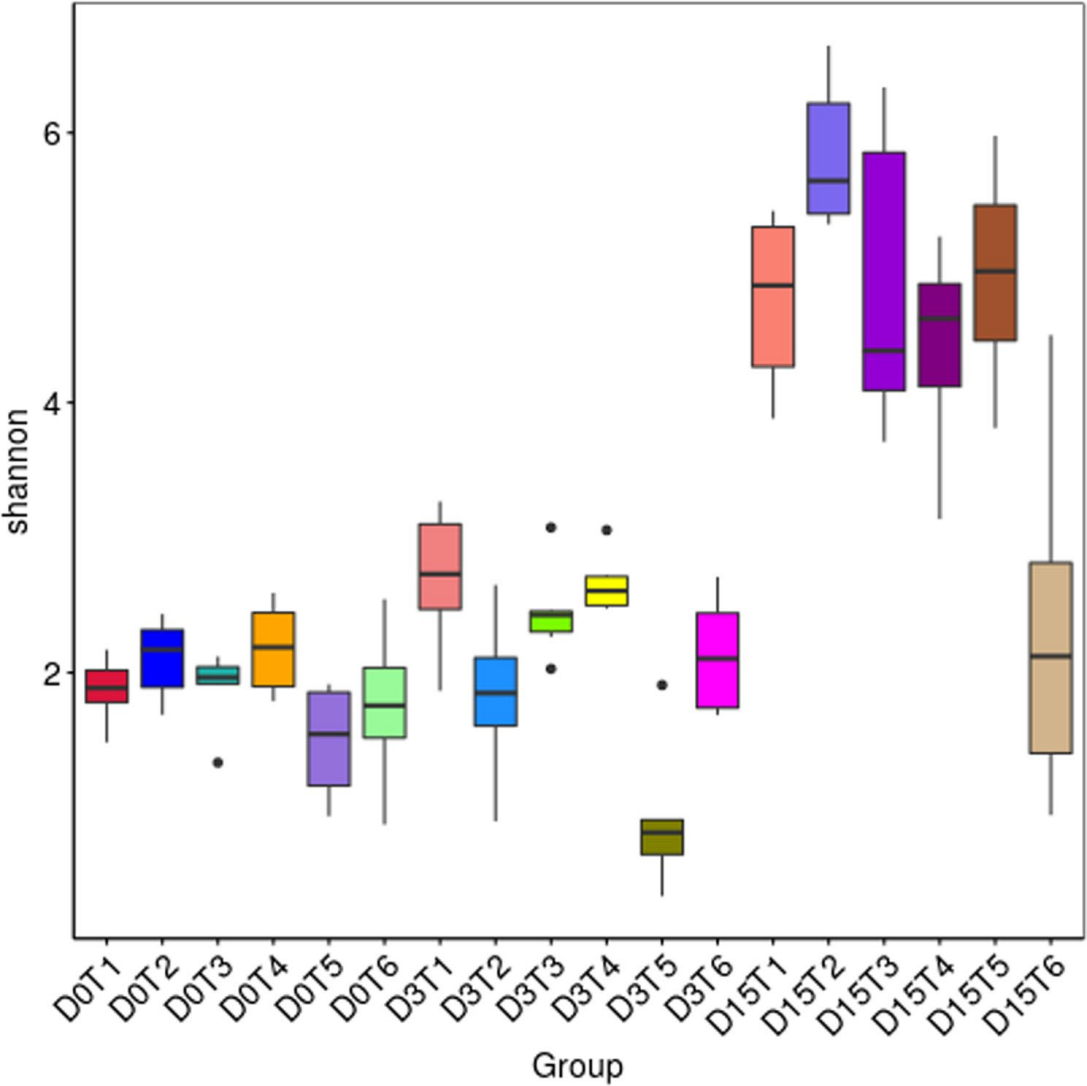
Shannon diversity index of gut microbiota across treatment groups at day 1 (D0T), day 7 (D3T), and day 42 (D15). 1 – low-dose, 2 – medium-dose, 3 – positive control, 4 – high-dose, 5 – enrofloxacin, 6 – negative control

The Shannon index showed clear temporal shifts in community composition (Fig. 1). At day 1 (pre-challenge), between-group differences were not statistically significant (Kruskal–Wallis H(5) = 9.712, *p* = 0.0838). At day 7, Shannon diversity differed significantly among groups (H(5) = 20.538, *p* = 0.00099). Dunn–FDR post hoc tests indicated lower Shannon diversity in the enrofloxacin group compared with the low-dose (adjusted *p* = 0.00224), high-dose (adjusted *p* = 0.00224), and positive control groups (adjusted *p* = 0.0393). By day 42, Shannon diversity also differed among groups (H(5) = 16.516, *p* = 0.00552), with the medium-dose group showing higher Shannon diversity than the negative control group (adjusted *p* = 0.00118).

**Fig. 2:**
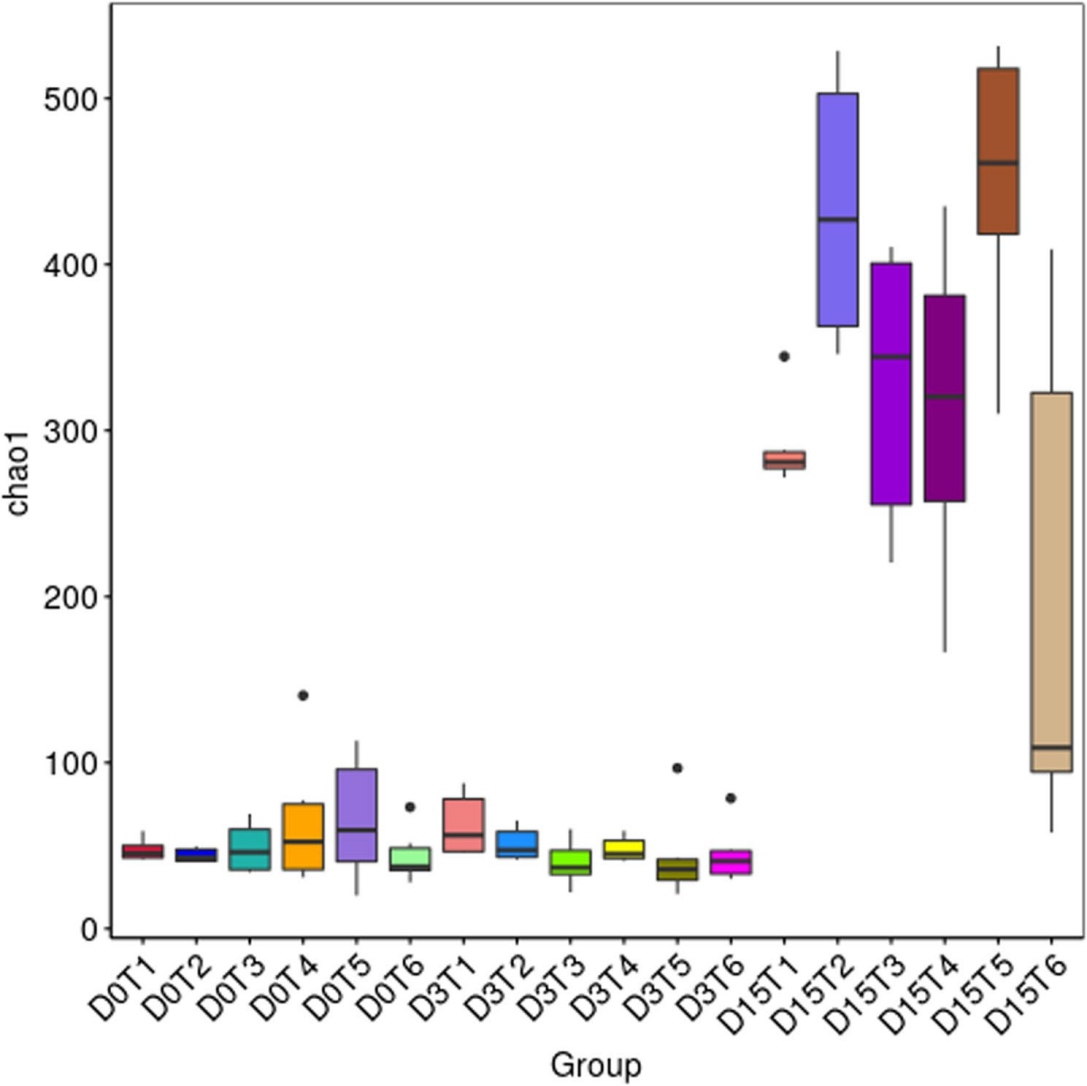
Chao1 richness index of gut microbiota across treatment groups at day 1 (D0T), day 7 (D3T), and day 42 (D15). 1 – low-dose, 2 – medium-dose, 3 – positive control, 4 – high-dose, 5 – enrofloxacin, 6 – negative control

The Chao1 index revealed low species richness on days 1 and 7 across all groups (Fig. 2). Chao1 richness did not show significant between-group differences at day 1 (H(5) = 2.469, *p* = 0.781) or day 7 (H(5) = 9.308, *p* = 0.0974), but differed significantly at day 42 (H(5) = 15.298, *p* = 0.00916). At day 42, Dunn–FDR post hoc testing indicated higher Chao1 richness in the enrofloxacin group compared with the negative control (adjusted *p* = 0.0374), lower richness in the low-dose group compared with enrofloxacin (adjusted *p* = 0.0471), and higher richness in the medium-dose group compared with the negative control (adjusted *p* = 0.0473). Similarly, observed feature richness did not differ significantly at day 1 (H(5) = 1.677, *p* = 0.892) or day 7 (H(5) = 9.885, *p* = 0.0785), but differed at day 42 (H(5) = 15.117, *p* = 0.00988), with higher observed features in the enrofloxacin group compared with the negative control (adjusted *p* = 0.0422) and lower observed features in the low-dose group compared with enrofloxacin (adjusted *p* = 0.0471).

Additional α-diversity metrics (Dominance, Observed features, Pielou’s evenness, Simpson index) are presented in the Supplementary Material (Figs. S2–S5). These indices confirmed the trends observed with Shannon and Chao1: by day 42, all treatment groups developed significantly richer and more balanced microbial communities, whereas the negative control consistently displayed lower values. Enrofloxacin treatment caused a pronounced early reduction in richness and evenness, reflected in high dominance scores and low Pielou’s evenness, although partial recovery was evident by the end of the trial.

On day 1 (pre-challenge), differences between groups were moderate. The highest species richness (Chao1, Observed features) was detected in the high-dose fenugreek and enrofloxacin groups, while the lowest values occurred in the medium-dose and negative control groups. Shannon and Simpson indices suggested that medium and high fenugreek doses supported more diverse and evenly distributed communities, in contrast to the enrofloxacin group, where a few taxa dominated.

By day 7, diversity increased in most groups compared to baseline. Low- and high-dose fenugreek supplementation produced the highest Shannon and Simpson indices, reflecting richer and more balanced communities, whereas enrofloxacin treatment resulted in the lowest values, consistent with antibiotic-driven disruption and dominance of a few taxa.

By day 42, all groups exhibited markedly higher richness and diversity compared to earlier time points. The medium-dose phytobiotic treated group achieved the highest diversity indices, indicating the most balanced microbial structure, while low- and high-dose treatments also supported stable communities. Enrofloxacin-treated birds showed moderate recovery, whereas the negative control displayed variable outcomes, with some samples maintaining relatively low richness.

### Beta diversity

Beta diversity analyses indicated pronounced temporal restructuring of gut microbial communities and treatment-associated separation. Weighted UniFrac distance heatmaps (Supplementary Fig. S6) suggested that Day 1 (pre-challenge) and Day 7 profiles were more similar to each other than to Day 42, consistent with age-driven community maturation.

Distance-based multivariate statistics on Bray–Curtis dissimilarities supported these patterns. Across timepoints, all pairwise PERMANOVA (Adonis) comparisons were significant (median R² = 0.494, range 0.305–0.823; BH–FDR-adjusted *p* ≤ 0.035; Supplementary Table S8), indicating strong temporal shifts (e.g., D0T2 vs. D15T2: R² = 0.743, *p* = 0.003, BH–FDR *p* = 0.0065). Within individual timepoints, 26/45 treatment contrasts were significant (median R² = 0.295, range 0.011–0.811; Supplementary Table S9), with the most pronounced separation on Day 7, particularly involving the enrofloxacin group (e.g., D3T4 vs. D3T5: R² = 0.811, *p* = 0.002, BH–FDR *p* = 0.0065; D3T1 vs. D3T5: R² = 0.665, *p* = 0.001, BH–FDR *p* = 0.0065). At Day 42, the medium-dose group remained clearly separated from the negative control (D15T2 vs. D15T6: R² = 0.551, *p* = 0.003, BH–FDR *p* = 0.0065).

These findings were concordant with ANOSIM and MRPP, which also indicated significant separation across timepoints and for multiple within-timepoint treatment contrasts (Supplementary Tables S10–S11) (e.g., ANOSIM D3T4 vs. D3T5: *R* = 1.000, *p* = 0.001, BH–FDR *p* = 0.0066; MRPP D3T4 vs. D3T5: A = 0.538, *p* = 0.003, BH–FDR *p* = 0.0067).

**Fig. 3:**
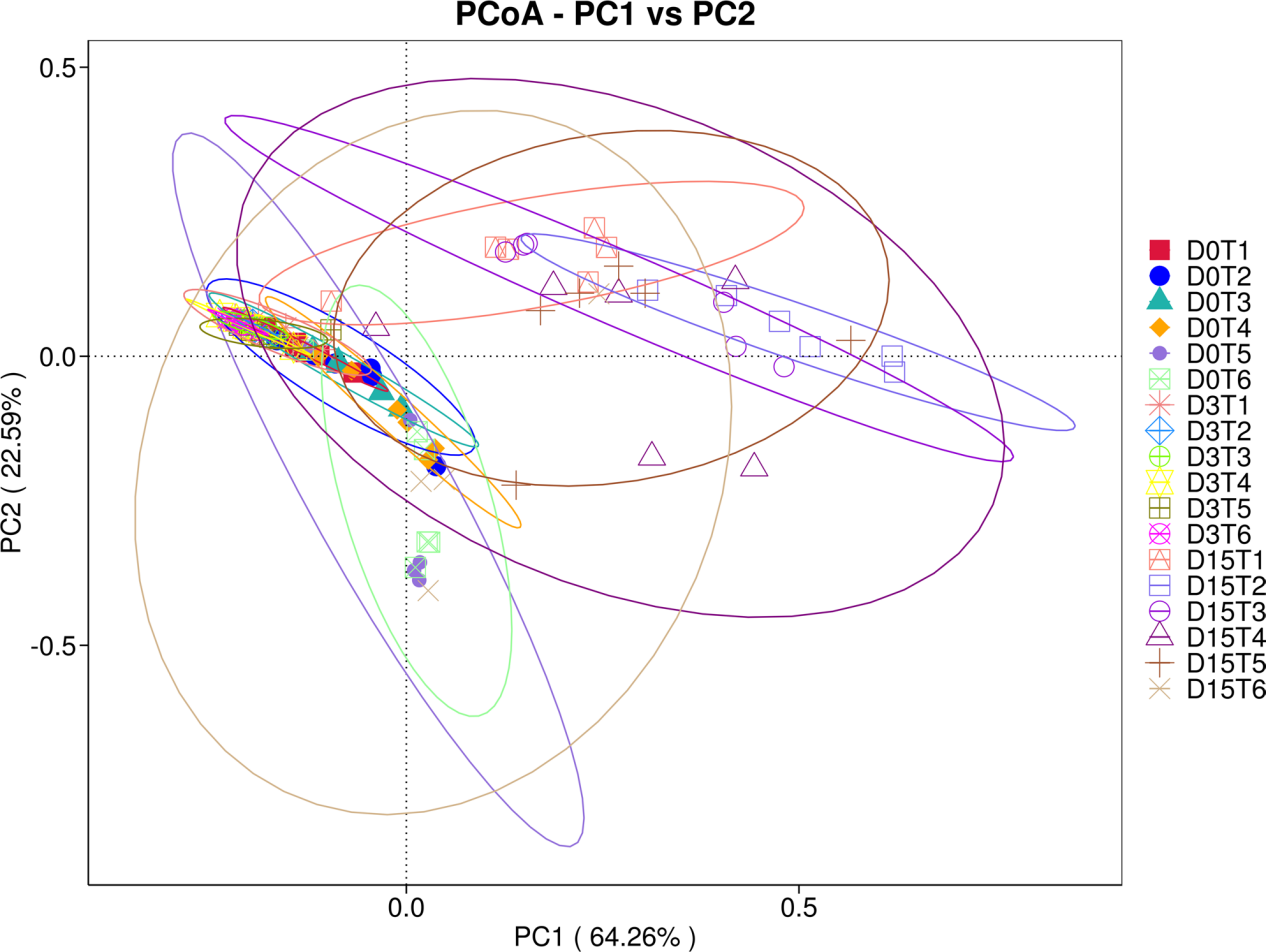
Principal Coordinates Analysis (PCoA) of gut microbiota based on weighted UniFrac distances (PC1 vs. PC2). Each point represents one pooled sample, coloured by group–timepoint category (D0T1–D15T6). Ellipses indicate 95% confidence regions around group centroids to aid visual interpretation. The proportion of variance explained by each axis is shown in parentheses on the axes (PC1: 64.26%, PC2: 22.59%). Weighted UniFrac incorporates phylogenetic relatedness and relative abundance, emphasizing shifts in dominant taxa across time points and treatments

**Fig. 4:**
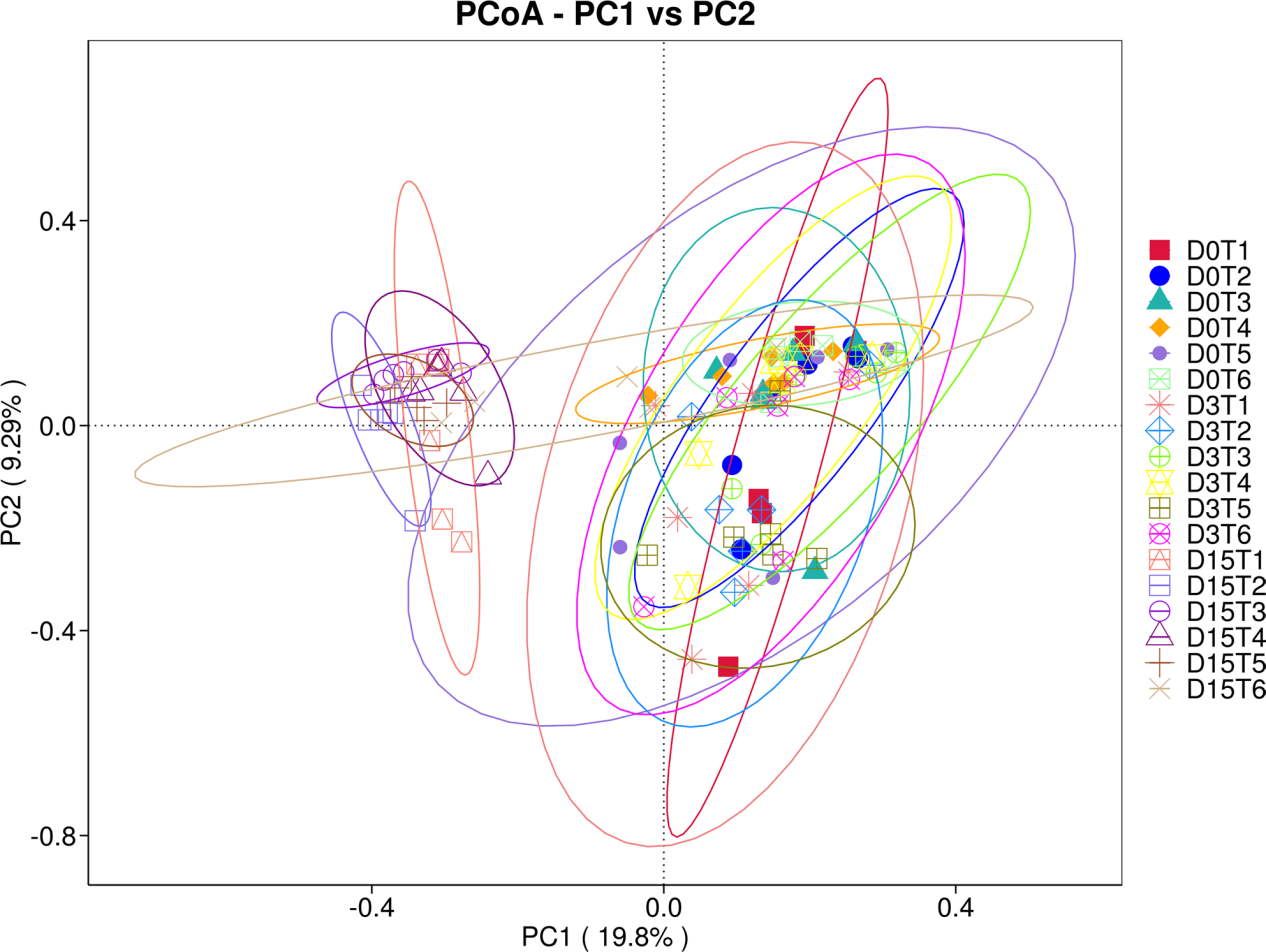
Principal Coordinates Analysis (PCoA) of gut microbiota based on unweighted UniFrac distances (PC1 vs. PC2). Each point represents one pooled sample, coloured by group–timepoint category (D0T1–D15T6). Ellipses indicate 95% confidence regions around group centroids. The proportion of variance explained by each axis is shown in parentheses on the axes (PC1: 19.8%, PC2: 9.28%). Unweighted UniFrac is based on presence/absence and phylogenetic relatedness, highlighting differences driven by low-abundance taxa and compositional turnover

Principal Coordinates Analysis (PCoA) supported the observed separation of communities across time and treatments. In line with the ordination patterns, PERMANOVA confirmed significant differences in community composition using Weighted UniFrac (R² = 0.418, *p* = 0.001) and Unweighted UniFrac (R² = 0.312, *p* = 0.003), indicating that both sampling time and treatment contributed to the observed clustering (Figs. 3 and 4).

To provide formal statistical support for the ordination, we performed permutation-based PERMANOVA on the same distance matrix. Time point explained most of the observed variation and was highly significant (pseudo-F = 23.10, R² = 0.755, *p* = 0.001; 999 permutations), consistent with the pronounced displacement of day 42 communities in the PCoA space. In contrast, when samples were grouped by treatment irrespective of time, the overall treatment effect was small and not significant (pseudo-F = 0.287, R² = 0.107, *p* = 0.961), indicating that temporal progression dominated the community-wide signal, while treatment-related differences were comparatively subtle and are best interpreted in conjunction with the complementary multivariate beta-diversity tests reported above.

Hierarchical clustering using UPGMA (Supplementary Fig. S7) is presented as an exploratory visualization and suggested that samples clustered primarily by time point, with Day 1 (pre-challenge) and Day 7 profiles grouping more closely and Day 42 forming distinct clusters. To statistically evaluate phylum-level compositional balance, we calculated the Firmicutes-to-Bacteroidota (F/B) ratio for each sample using phylum-level read counts with a pseudocount (Firmicutes + 1) / (Bacteroidota + 1) and performed non-parametric comparisons across treatments within each time point. The log10-transformed F/B ratio differed significantly across treatments at Day 42 (Kruskal–Wallis H = 11.224, *p* = 0.047), whereas no overall differences were detected at Day 1 or Day 7 (both *p* ≥ 0.18). Post hoc Dunn testing with BH–FDR correction at Day 42 showed that the negative control exhibited a markedly higher log10(F/B) ratio (median 3.32) compared with the low-dose (median − 0.46; q = 0.0299) and medium-dose phytobiotic groups (median − 0.42; q = 0.0299). Full test statistics and pairwise comparisons are provided in Supplementary Table S12-S13.

Non-metric multidimensional scaling (NMDS) analysis corroborated these results (Supplementary Fig. S8), showing clear segregation of day 42 samples, particularly in treated groups. Low stress values (0.053 and 0.18) confirmed the robustness of these ordinations. PCoA and NMDS were included as complementary visualizations of beta-diversity under different ordination assumptions; formal statistical inference was based on permutation-based tests (PERMANOVA/ANOSIM/MRPP), not on ordination plots.

### Gut microbiota dynamics at phylum level

**Fig. 5:**
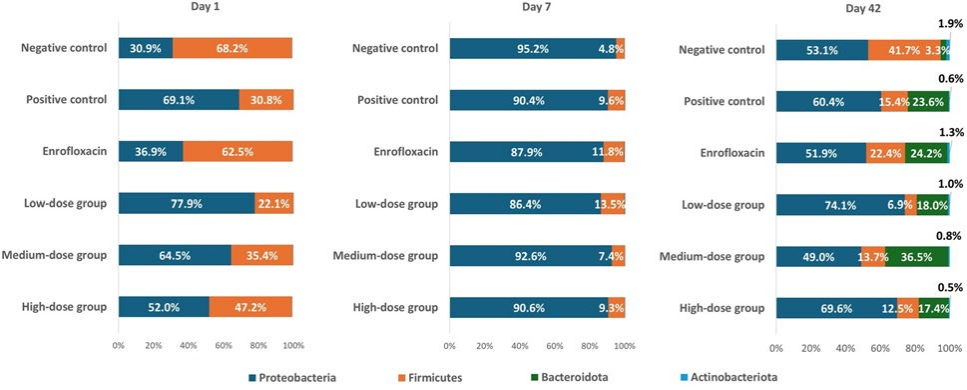
Phylum-level composition of the gut microbiota in *Salmonella* and *Escherichia coli*-infected chickens supplemented with phytobiotic at different doses, enrofloxacin, or control diets, sampled on day 1, day 7, and day 42

At baseline (day 1), microbial communities were already heterogeneous across groups (Fig. 5). The dominant phyla were Proteobacteria and Firmicutes, but their relative proportions varied: the negative control harbored a Firmicutes-rich profile (68% Firmicutes, 31% Proteobacteria), whereas the positive control and several treatment groups displayed the opposite trend, with Proteobacteria exceeding 60%. These early differences reflect natural variability in microbial colonization.

Following infection (day 7), communities underwent a profound shift, characterized by an overwhelming expansion of Proteobacteria (> 85%) and a marked reduction of Firmicutes (5–15%). This pattern is consistent with acute dysbiosis triggered by *Salmonella* and *E. coli* challenge, where Gammaproteobacteria rapidly dominate at the expense of commensal taxa.

By day 42, partial recovery of community balance was observed. Notably, the medium-dose phytobiotic group exhibited the most pronounced restructuring, with increased Bacteroidota and reduced Proteobacteria, indicating a shift towards a more diverse and stable microbiota. In contrast, low- and high-dose phytobiotic treatments showed limited restorative effects. The enrofloxacin group largely resembled the positive control, suggesting that antibiotic treatment alone did not re-establish a balanced microbiota.

### Gut microbiota dynamics at the class level

**Fig. 6:**
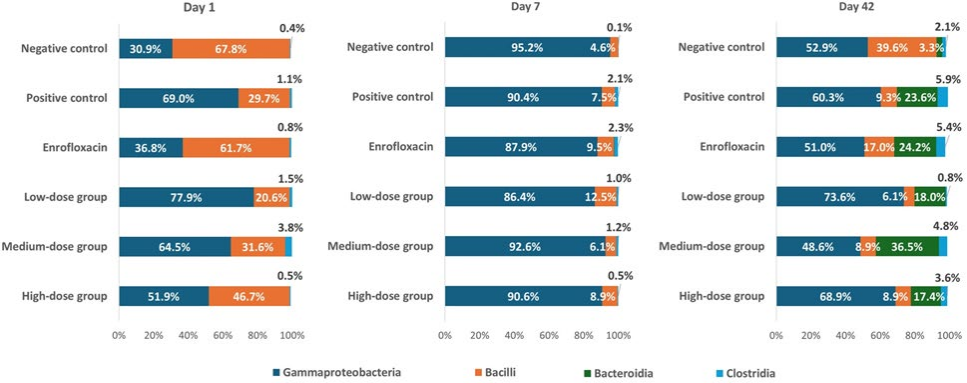
Class-level composition of the gut microbiota in *Salmonella* and *Escherichia coli*-infected chickens supplemented with fenugreek extract at different doses, enrofloxacin, or control diets, sampled on day 1, day 7, and day 42

At baseline (day 1), community composition varied markedly between groups (Fig. 6). In the negative control, Bacilli dominated (67.8%), with moderate proportions of Gammaproteobacteria (30.9%) and minimal Clostridia, suggesting a more balanced initial state. In contrast, positive control was characterized by a reverse profile, with Gammaproteobacteria predominance (69.0%) and reduced Bacilli (29.7%). Most other groups displayed varying degrees of Gammaproteobacteria dominance, reflecting early heterogeneity in colonization.

By day 7, the impact of infection was evident: Gammaproteobacteria became overwhelmingly dominant across all groups, indicating acute dysbiosis. At this stage, treatments did not yield pronounced differences, underscoring that neither fenugreek supplementation nor enrofloxacin was able to prevent pathogen-driven disruption of the microbial community.

By day 42, community composition shifted towards partial recovery. The medium-dose fenugreek group exhibited the most pronounced rebalancing, with increased Bacteroidia and reduced Gammaproteobacteria, pointing to a more resilient and diverse microbial structure. Low- and high-dose fenugreek were less effective, while the enrofloxacin group remained similar to the positive control, failing to fully restore Bacilli–Gammaproteobacteria balance.

### Gut microbiota dynamics at the order level

**Fig. 7:**
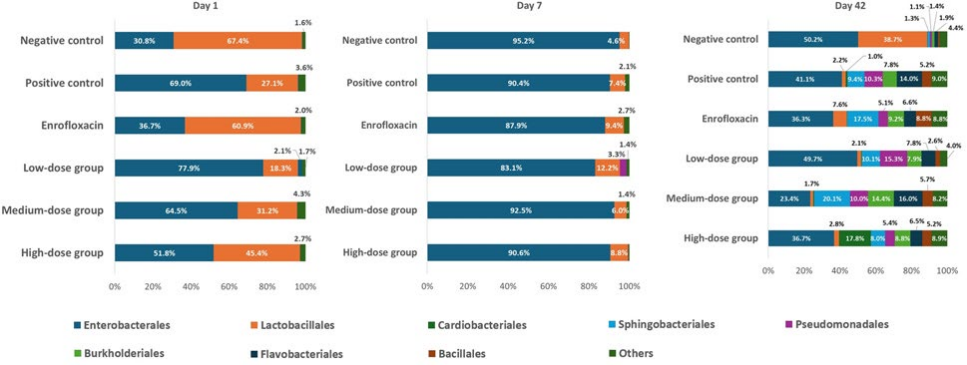
Order-level composition of the gut microbiota in *Salmonella* and *Escherichia coli*-infected chickens supplemented with phytobiotics at different doses, enrofloxacin, or control diets, sampled on day 1, day 7, and day 42

At the order level (Fig. 7), microbial communities on day 1 (pre-challenge) were primarily dominated by Enterobacterales and Lactobacillales. The negative control group displayed a more balanced profile with higher Lactobacillales (67.4%), whereas the positive control and low-dose phytobiotic groups were characterized by strong Enterobacterales dominance (> 70%). The enrofloxacin group exhibited a more intermediate composition, with both orders coexisting at comparable levels.

By day 7, a marked homogenization occurred across all groups: Enterobacterales became overwhelmingly dominant (83–95%), while Lactobacillales dropped to below 10%. This shift reflected severe dysbiosis and loss of diversity, with community structure approaching collapse. Neither antibiotic treatment nor phytobiotic supplementation was able to prevent this acute pathogen-driven disruption.

By day 42, community diversity partially recovered. The relative abundance of Enterobacterales decreased in most groups, while several other orders — including Sphingobacteriales, Pseudomonadales, Flavobacteriales, Bacillales, and Burkholderiales — emerged in notable proportions. The enrofloxacin group showed the highest order-level diversity, likely reflecting secondary colonization processes. Importantly, the medium-dose phytobiotic group exhibited the strongest rebalancing effect, with Enterobacterales reduced to 23.4% and multiple other orders present at moderate abundances (8–20%). These findings suggest that medium-dose supplementation promoted a more resilient and diverse microbial community compared to both low- and high-dose treatments.

### Gut microbiota dynamics at the family level

**Fig. 8:**
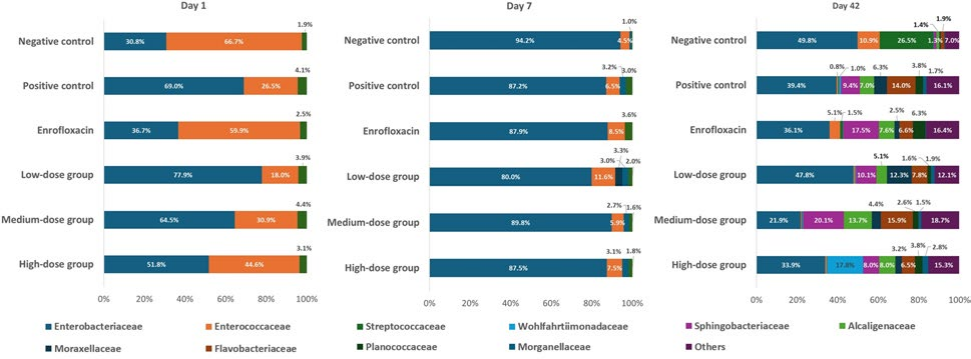
Family-level composition of the gut microbiota in *Salmonella* and *Escherichia coli*-infected chickens supplemented with phytobiotic feed additive at different doses, enrofloxacin, or control diets, sampled on day 1, day 7, and day 42

At the family level (Fig. 8), early communities (day 1) were dominated by Enterococcaceae and Enterobacteriaceae, with the negative control group showing higher Enterococcaceae abundance (67.7%), reflecting differences in baseline community structure; given the heterogeneity within *Enterococcus* spp., we avoid health-related inference from Enterococcaceae abundance alone. In contrast, the positive control and the low- and medium-dose phytobiotic groups were dominated by Enterobacteriaceae (> 64%), suggesting an early shift towards opportunistic taxa. The enrofloxacin and high-dose groups displayed more balanced proportions of Enterobacteriaceae and Enterococcaceae.

By day 7, Enterobacteriaceae became almost exclusively dominant (80–95%) across all groups, while Enterococcaceae declined sharply to < 12%. Other families such as Moraxellaceae and Streptococcaceae nearly disappeared, reflecting severe antibiotic- and infection-driven dysbiosis and a collapse of community complexity.

By day 42, microbial communities diversified again, but recovery patterns differed across treatments. In the negative control, Enterobacteriaceae (49.8%) coexisted with Streptococcaceae (26.5%). The positive control exhibited a more heterogeneous community with several families (Flavobacteriaceae, Sphingobacteriaceae, Morganellaceae, Planococcaceae) present at moderate levels. Enrofloxacin treatment resulted in high relative abundances of Enterobacteriaceae (36.1%) alongside Flavobacteriaceae and Sphingobacteriaceae, suggesting secondary colonization. Low- and high-dose phytobiotic groups showed partial recovery with moderate diversity. Strikingly, the medium-dose phytobiotic group demonstrated the richest and most balanced family-level composition, with Enterobacteriaceae reduced to 21.9% and multiple families (Flavobacteriaceae, Sphingobacteriaceae, Morganellaceae, Planococcaceae) each contributing 8–20%.

### Gut microbiota dynamics at the genus level

**Fig. 9:**
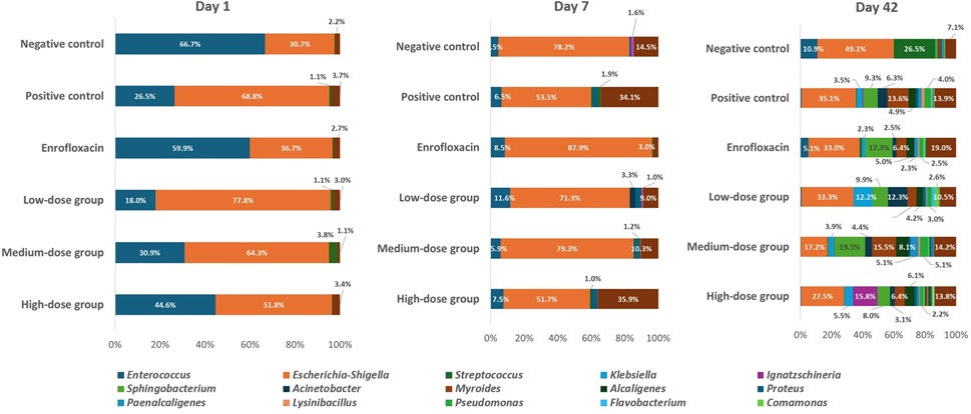
Genus-level composition of the gut microbiota in *Salmonella* and *Escherichia coli*-infected chickens supplemented with phytobiotic feed additive at different doses, enrofloxacin, or control diets, sampled on day 1, day 7, and day 42. Only genera with ≥ 1% relative abundance are shown; low-abundance taxa (including *Salmonella*) are not displayed

At the genus level (Fig. 9), Day 1 (pre-challenge) cloacal communities were dominated by Enterococcus and *Escherichia–Shigella*. The negative and enrofloxacin control groups showed higher relative abundance of Enterococcus, whereas the positive control group displayed increased Escherichia–Shigella. Given the taxonomic limitations of short-amplicon 16 S profiling and the heterogeneity within *Enterococcus* spp. (including opportunistic and pathogenic lineages such as *Enterococcus cecorum*), we avoid interpreting *Enterococcus* enrichment as a direct indicator of “health” and instead describe these patterns as differences in baseline cloacal community structure. Accordingly, genus-level trends are interpreted cautiously and used primarily to identify lineages contributing to the community-wide separation supported by permutation-based beta-diversity statistics.

By day 7, *Escherichia–Shigella* expanded dramatically across nearly all groups, exceeding 78.2% in the negative control, 87.9% in the enrofloxacin group, and 79.3% in the medium-dose phytobiotic group. *Enterococcus* was strongly suppressed in all treatments. In some groups (e.g., positive control, high-dose), opportunistic genera such as *Myroides* also emerged, reflecting acute dysbiosis under infection and treatment pressure.

By day 42, genus-level diversity markedly increased, with 7–10 genera contributing to community structure in most groups. In the negative control, *Escherichia–Shigella* decreased to 49.1%, while *Comamonas* (26.5%) and *Enterococcus* (10.9%) re-emerged. The positive control showed a highly heterogeneous profile including *Acinetobacter*, *Proteus*, and *Comamonas*. The enrofloxacin group exhibited reduced *Escherichia–Shigella* (33.0%) but an overgrowth of *Paenacaligenes* and *Pseudomonas*, suggesting antibiotic-driven secondary colonization. Low-dose phytobiotic treatment produced a more balanced community (*Escherichia–Shigella* 33.3%, *Enterococcus* 12.2%, *Paenacaligenes* 12.3%, *Comamonas* 10.5%). Notably, medium-dose phytobiotic supplementation yielded the richest genus-level diversity, with *Paenacaligenes* (19.5%) and *Enterococcus* (17.2%) as key contributors. The high-dose group also showed reduced *Escherichia–Shigella* (27.5%) and increased *Paenacaligenes* (15.8%), alongside several low-abundance genera.

In addition, we constructed a phylogenetic tree of the 100 most abundant genera, with relative abundances mapped onto the branches (Supplementary Fig. S9). This analysis confirmed that the communities were primarily structured by dominant members of Firmicutes and Proteobacteria, forming the backbone of the gut microbiota across treatments and time points.

## Discussion

Our study shows that phytobiotic feed supplementation (fenugreek and turmeric extracts) modulated cloacal microbiota structure in chickens within a controlled dual-challenge colonization model, as reflected by consistent shifts in alpha diversity indices and beta-diversity separation across time points and treatment groups. Notably, the medium dose was associated with the most balanced community profile at the end of the trial, whereas enrofloxacin exposure was linked to the strongest early community disruption (i.e., reduced alpha diversity and altered community structure) in the post-challenge period. Because clinical outcomes (e.g., performance metrics) were not a primary endpoint of this microbiome-focused study, we interpret these findings as community-level responses to the applied experimental conditions rather than as direct evidence of disease severity or “pathogen overgrowth”; where relevant, we describe changes conservatively as relative enrichment of Enterobacterales/Enterobacteriaceae (including the *Escherichia–Shigella* group) rather than as clinical colibacillosis [19, 51]. Importantly, not all *E. coli* strains are pathogenic, and 16 S rRNA amplicon sequencing cannot resolve virulence at strain level. Accordingly, we interpret changes attributed to *E. coli* primarily as shifts in Enterobacterales and Enterobacteriaceae abundance rather than as direct evidence of colibacillosis. While short-amplicon 16 S rRNA sequencing has limited sensitivity for detecting low-abundance *Salmonella*, bacteriological re-isolation provided independent confirmation of successful colonization. Accordingly, microbiota analyses focused on community-level responses to the applied challenge rather than direct quantification of *Salmonella* loads. Bacteriology was used to confirm colonization qualitatively; however, quantitative enumeration was not performed, thus treatment effects on *Salmonella* load cannot be inferred from culture data. A limitation of the present 16 S rRNA gene amplicon workflow is the use of 30 PCR cycles. Although a high-fidelity polymerase was applied, higher cycle numbers may increase amplification bias and propagate low-frequency errors, potentially affecting relative abundance estimates for some taxa. In addition, the study did not include a sequenced no-template PCR control and a mock community standard, which would provide a more direct assessment of contamination and taxonomic assignment performance. Future studies will incorporate a sequenced no-template PCR control and a defined mock community standard to further benchmark potential contamination and taxonomic assignment performance within the same workflow. These limitations should be considered when interpreting taxon-level shifts; however, the sample matrix represents a high-biomass gastrointestinal source, and the primary conclusions are drawn from consistent community-level patterns across groups processed in an identical manner. Because short-amplicon 16 S rRNA profiling has limited resolution below the genus level for several Enterobacteriaceae lineages, we interpret taxonomic shifts primarily at genus (or higher) level (e.g., the *Escherichia/Shigella* group) rather than as species-level changes. A mock community control was not included in the sequencing run; therefore, genus-level taxonomic assignments should be interpreted conservatively. To mitigate this limitation, we applied stringent quality filtering and chimera removal and report results primarily at ranks where 16 S amplicon profiling is most reliable. Species-level interpretation is avoided, and ambiguous taxa (e.g., Escherichia–Shigella) are reported in the standard combined form.

On day 1 (pre-challenge), alpha diversity indices (Shannon and Simpson) indicated that medium and high doses of phytobiotic enhanced community evenness and richness, suggesting an early stabilization of gut colonization. This aligns with Paneru et al. [20], who reported increased *Firmicutes* and beneficial genera such as *Alistipes*, *Bacteroides* and *Prevotellaceae* in fenugreek-supplemented broilers [20, 22, 51]. By day 7, both low and high doses further promoted diversity, whereas the enrofloxacin group displayed markedly reduced values, reflecting the well-documented disruptive impact of antibiotics on microbiota [19, 51]. At the end of the experiment (day 42), the medium dose yielded the highest species richness and evenness, consistent with long-term microbiome stabilization and partly mirroring Paneru et al. [22], who observed improved diversity and immune modulation after 42 days of fenugreek feeding [22]. In contrast, the antibiotic group showed only partial recovery, and the negative control revealed substantial individual variability, indicating that without intervention the gut community remains less stable.

The consistently lower Shannon diversity observed in the negative control at certain time points is notable and may reflect biological rather than technical factors. Early-life chicken gut communities can exhibit substantial inter-individual variability and stochastic colonization dynamics, and the absence of an infection-driven perturbation may result in a less diverse but more uneven community dominated by a limited number of early colonizers. In addition, cage- and environment-associated effects (e.g., microenvironment, litter exposure, and social mixing) can contribute to between-group differences in community assembly even under standardized husbandry. Importantly, all samples were collected and processed under an identical protocol, which reduces the likelihood that this pattern is driven by systematic pre-analytical handling differences. Nevertheless, because no clinical scoring or performance metrics were recorded, and 16 S profiling does not directly measure disease status, we interpret the low diversity in the negative control cautiously as a group-specific community assembly pattern rather than evidence of pathology.

Collectively, these results underscore the dose-dependent microbiome-modulating potential of the combination of fenugreek and turmeric, particularly at medium concentration, as a natural alternative to antibiotics for maintaining gut homeostasis in poultry.

At baseline (Day 1), cloacal microbiota profiles reflected early post-hatch colonization and were dominated by Firmicutes and Proteobacteria, consistent with the rapid and variable establishment of microbial communities in newly hatched chicks. The negative control showed higher relative abundance of Bacilli (including *Enterococcus*), whereas the positive control displayed higher relative abundance of Enterobacterales/Enterobacteriaceae, reported in the standard combined form as the *Escherichia–Shigella* group. Because Enterococcus spp. and *Escherichia/Shigella*-related taxa are heterogeneous and short-amplicon 16 S profiling does not resolve strain-level pathogenicity, we avoid assigning “healthy” versus “dysbiotic” labels to these baseline configurations and interpret them as between-group differences in initial community structure [52]. Such early heterogeneity is well recognized in poultry, where substantial variation can arise within hours post-hatch due to biological, environmental, and management factors [53, 54]. These initial differences are important, as baseline community structure may influence subsequent microbiota trajectories and host responses to challenge.

By day 7, infection induced a dramatic community shift characterized by the explosive expansion of *Proteobacteria* – particularly *Gammaproteobacteria* and Enterobacterales, accompanied by the near disappearance of beneficial Gram-positive taxa such as *Enterococcus*. This profile is emblematic of enteric challenge–associated dysbiosis, where the altered gut environment can favor the outgrowth of *Gammaproteobacteria* with concomitant reduction in overall community diversity [55, 56]. Notably, even enrofloxacin treatment failed to restore microbial balance at this stage, with *Proteobacteria* still accounting for nearly 88% of the community. Similarly, phytobiotic supplementation did not prevent the dominance of *Escherichia–Shigella*, underscoring that early post-infection dysbiosis represents a particularly refractory window to therapeutic modulation. Family-level analysis further highlighted the strong selective pressure of antibiotics, leading to reduced biodiversity and a shift toward Gram-negative opportunists [57, 58]. Together, these findings emphasize that interventions in the acute phase may have limited efficacy, and microbiome restoration is more likely achievable during later recovery.

By day 42, the gut microbiota showed substantial restructuring compared with day 7, reflecting the onset of recovery from infection. The dominance of *Escherichia–Shigella* diminished, while *Firmicutes* and *Bacteroidota* rebounded, and community diversity expanded with the emergence of *Pseudomonadales*, *Burkholderiales* and other taxa. Such age-dependent stabilization of the chicken microbiome, typically occurring by 4–6 weeks of age, has been consistently reported in broiler studies [34, 59]. Treatment-specific effects were evident: medium-dose phytobiotic yielded the most balanced community structure, while antibiotic-treated birds developed an alternative state characterized by persistent colonization of opportunists such as *Pseudomonas*. This is consistent with previous reports showing that antibiotics can induce long-term shifts in microbial composition, promoting secondary colonization [53, 60]. Interestingly, effects of the combination of fenugreek and turmeric appeared dose-dependent but non-linear – while low doses had minimal impact and high doses favored alternative profiles, medium dosing facilitated the most robust recovery. Such biphasic responses are well described for phytogenic, reflecting their complex bioactivity [61, 62]. The Day 42 community composition may appear different from profiles commonly reported for cecal contents in broilers, where Lachnospiraceae and Ruminococcaceae (and often Lactobacillaceae) are frequently dominant. In our study, microbiota profiling was based on cloacal swabs rather than intestinal segment contents, and cloacal-associated communities can differ from cecal microbiota due to anatomical location, exposure to the external environment, and distinct substrate availability. In addition, age-related maturation, infection challenge, and antimicrobial/phytobiotic interventions can shift community structure, and our pooled-sample design may accentuate consistent group-level signals while attenuating inter-individual variation. Therefore, we interpret the Day 42 taxonomic profile as representative of the distal gut/cloacal microbiota under the applied experimental conditions rather than as a direct surrogate of cecal community composition.

Taxonomic interpretation was intentionally structured across ranks to couple broad community shifts (phylum/class) with lineage-level drivers (family/genus), while species-level inferences from short-amplicon 16 S were treated cautiously. Across groups, and particularly in non-treated birds, we observed a microbiota profile that may appear atypical when compared with an “idealized” reference community. Such deviations can arise from biological and micro-environmental heterogeneity that is common in poultry populations, especially during early-life community assembly and under field-like housing conditions. Pen-level exposures (e.g., litter contact, microclimate, feed and water intake patterns), host-to-host variation, and stochastic colonization dynamics may transiently favor Enterobacterales-dominated configurations and reduced evenness in a subset of birds. Importantly, because all groups were sampled and processed under identical conditions, the observed baseline variability is interpreted as biological rather than technical, and it does not undermine the main conclusions, which are supported by consistent dose-dependent patterns and concordant community-level metrics across the experimental groups.

Our findings align with and extend earlier studies on fenugreek. Paneru et al. [20] reported shifts towards higher *Firmicutes* and enrichment of beneficial genera (e.g. *Alistipes*, *Bacteroides*, *Prevotellaceae*) in fenugreek-supplemented broilers [20]. Yang et al. [25] found improved α-diversity and reductions in pathogens such as *Campylobacter* with fenugreek extract [25], while Fawaz et al. [24] highlighted antimicrobial effects of fenugreek oil, including decreased *E. coli* and *Salmonella* loads alongside higher *Lactobacillus* abundance [24]. Together, these results underscore that fenugreek – whether seed powder, extract, or essential oil – consistently supports microbiome modulation and pathogen suppression, though outcomes vary with dose and formulation.

Overall, our study demonstrates that phytobiotic supplementation of the combination of fenugreek and turmeric, particularly at medium doses, promotes long-term microbial diversity and community balance, contrasting with the dysbiosis and alternative colonization patterns induced by antibiotics. These results strengthen the evidence that phytogenic additives can act as sustainable microbiome stabilizers in poultry production, reducing reliance on antibiotics and their associated public health risks. This experiment was primarily designed to characterize microbiota-level responses to the applied challenge and interventions. Clinical outcomes and production performance parameters were not pre-specified endpoints; therefore, conclusions are restricted to community composition and diversity rather than clinical efficacy. We acknowledge that extraction blanks, PCR negatives and mock community controls were not included in the original sequencing batch. To minimize contamination risk, samples were processed using sterile, separated workflows and identical handling across groups; thus, any low-level background contamination would be expected to bias comparisons towards similarity rather than producing consistent group-specific patterns. Nevertheless, the absence of dedicated controls is a limitation and should be considered when interpreting low-abundance taxa. Further validation across poultry breeds and management systems will be critical to establishing such phytobiotic products as a robust, scalable strategy for gut health management.

## Conclusion

Our study provides a comprehensive characterization of the effects of phytobiotic feed supplementation on the chicken gut microbiome under pathogen challenge. α-diversity metrics clearly demonstrated that supplementation – particularly at medium doses – enhanced microbial richness and balance, in contrast to the marked loss of diversity observed under enrofloxacin treatment. Beta-diversity and taxonomic analyses further confirmed the stabilizing role of fenugreek and turmeric, mitigating the dysbiosis triggered by infection and antibiotic exposure.

At the family and genus levels, the phytobiotic was associated with consistent community shifts, including reduced Enterobacterales/Enterobacteriaceae dominance at later time points and higher overall diversity compared with the infected untreated control.

Overall, our findings establish the combination of fenugreek and turmeric as a promising natural alternative to antibiotics in poultry, capable of supporting gut microbial equilibrium during infection. Given the dose-dependent but non-linear responses observed, future research should focus on optimizing dosing strategies and evaluating long-term ecological and health impacts across production systems.

## Supplementary Information

Supplementary Material 1.

## Data Availability

The raw data supporting the conclusions of this article will be made available by the corresponding author, without undue reservation. The datasets generated and/or analyzed during the current study are available in the National Library of Medicine: https://www.ncbi.nlm.nih.gov/bioproject/PRJNA1332038, accessed on 20 September 2025. Additional data is provided within the supplementary files.
