# Supplementary material for: Impact of plant-based antibiotic alternative supplemented feed on the gut microbiota of Bábolna Tetra-SL chickens experimentally infected with Salmonella enterica and Escherichia coli
Source: BMC Vet Res. 2026 Feb 28;22:204. doi: 10.1186/s12917-026-05381-3 (PMC13059556; doi:10.1186/s12917-026-05381-3)
Supplement: Supplementary file 1 — Supplementary Material 1. [file 12917_2026_5381_MOESM1_ESM.docx]

**Supplementary Table 1** The composition of the premix

| **Nutrient content** | | | |
| --- | --- | --- | --- |
| **Name** | **Value** | **Unit** |  |
| Dry Matter (DM) | 99.18 | % |  |
| Lysine | 4.53 | % |  |
| Methionine | 6.19 | % |  |
| Threonine | 3.92 | % |  |
| Calcium | 18.65 | % |  |
| Phosphorus | 7.38 | % |  |
| Sodium | 3.92 | % |  |
| Vitamin A, added | 250,000.00 | IU |  |
| Vitamin D3, added | 125,000.00 | IU |  |
| Vitamin E, added | 2 125.00 | mg |  |
| Choline chloride | 16,590.00 | mg |  |
| Niacin, added | 1 425.00 | mg |  |
| Pantothenic acid, added | 336.00 | mg |  |
| Vitamin B1, added | 90.00 | mg |  |
| Vitamin B12, added | 825.00 | mcg |  |
| Vitamin B2, added | 264.00 | mg |  |
| Vitamin B6, added | 144.00 | mg |  |
| Vitamin K3, added | 90.00 | mg |  |
| Biotin, added | 4 500.00 | mcg |  |
| Folic acid, added | 43.20 | mg |  |
| Zinc, added | 2 500.00 | mg |  |
| Iodine, added | 32.50 | mg |  |
| Manganese, added | 3 000.00 | mg |  |
| Copper, added | 387.50 | mg |  |
| Selenium, added | 7.50 | mg |  |
| Iron, added | 1 125.00 | mg |  |
| 6-Phytase | 13,000.00 | FTU |  |

**Supplementary Table 2** Composition of the additive in the feed.

| **Component name** | **Authorization category number** | **Authorization type** |
| --- | --- | --- |
| *Trigonella foenum graecum* extract | 2.b | Additive |
| Copper amino acid hydrate chelate | 3b406 | Additive |
| *Cichorium intybus* L. root | 4.4.1 | Raw material |
| *Curcuma longa* L.: extract | 2b | Additive |

**Supplementary Table 3** The groups are divided by treatment, with animals receiving pre-starter feed in weeks 1-3 and starter feed in weeks 4-6.

| **Group** | **Feed** | ***Salmonella* and *Escherichia coli* infection** | **Additive in the feed** |
| --- | --- | --- | --- |
|  |  |  |  |
| 1 | 1× dose | + | 0.1 g/kg |
| 2 |  | + |  |
| 3 |  | + |  |
| 4 | 10× dose | + | 1 g/kg |
| 5 |  | + |  |
| 6 |  | + |  |
| 7 | 100× dose | + | 10 g/kg |
| 8 |  | + |  |
| 9 |  | + |  |
| 10 | Antibiotic control | + | *Enrofloxacin |
| 11 |  | + |  |
| 12 |  | + |  |
| 13 | Positive control | + | - |
| 14 |  | + |  |
| 15 |  | + |  |
| 16 | Negative control | - | - |
| 17 |  | - |  |
| 18 |  | - |  |

* Baytril 100 mg/mL solution for mixing into drinking water A.U.V., at a dose of 10 mg/kg body weight mixed into drinking water for 5 days, according to the manufacturer's instructions.

**Supplementary Table 4** Summary of mortality and gross necropsy findings by experimental group.

| **Group** | **Initial**  **(n)** | **Final**  **(n)** | **Deaths (n)** | **Omphalitis (n)** | **Typhlitis (n)** | **Pale kidney (n)** | **Pinpoint grayish-white lesions (n)** |
| --- | --- | --- | --- | --- | --- | --- | --- |
| Low-dose | 45 | 37 | 8 | 2 | 2 | 3 | 1 |
| Medium-dose | 45 | 42 | 3 | 2 | 3 | 1 | 0 |
| High-dose | 45 | 37 | 8 | 3 | 3 | 1 | 1 |
| Antibiotic | 45 | 41 | 4 | 3 | 0 | 1 | 0 |
| Positive control | 45 | 34 | 11 | 6 | 5 | 3 | 2 |
| Negative control | 45 | 41 | 4 | 1 | 1 | 2 | 0 |

**Supplementary Table 5** Quality control statistics of 16S rRNA gene sequencing data

| **Sample** | **Group** | **Day** | **RawPE** | **Combined** | **Qualified** | **Nochime** | **Base(nt)** | **Avglen(nt)** | **GC** | **Q20** | **Q30** |
| --- | --- | --- | --- | --- | --- | --- | --- | --- | --- | --- | --- |
| A.1011 | Low-dose | Day 1 | 64662 | 64068 | 62895 | 60501 | 25927425 | 428.55 | 54.33% | 98.77% | 95.64% |
| A.1012 | Low-dose | Day 1 | 103960 | 102831 | 101069 | 98028 | 41940335 | 427.84 | 54.78% | 98.73% | 95.41% |
| A.1021 | Low-dose | Day 1 | 105478 | 104682 | 102678 | 98726 | 42314847 | 428.61 | 54.10% | 98.86% | 95.86% |
| A.1022 | Low-dose | Day 1 | 104588 | 103683 | 101757 | 98389 | 42178326 | 428.69 | 54.47% | 98.84% | 95.73% |
| A.1031 | Low-dose | Day 1 | 102455 | 101512 | 99729 | 93595 | 40148089 | 428.96 | 54.17% | 98.76% | 95.66% |
| A.1032 | Low-dose | Day 1 | 117005 | 115877 | 113755 | 105971 | 45456735 | 428.95 | 54.35% | 98.74% | 95.54% |
| A.1041 | Medium-dose | Day 1 | 102129 | 101270 | 99506 | 95406 | 40923630 | 428.94 | 54.26% | 98.80% | 95.64% |
| A.1042 | Medium-dose | Day 1 | 102166 | 101396 | 99452 | 95974 | 40927385 | 426.44 | 54.11% | 98.80% | 95.68% |
| A.1051 | Medium-dose | Day 1 | 102489 | 101651 | 99656 | 96855 | 41415672 | 427.60 | 53.32% | 98.77% | 95.58% |
| A.1052 | Medium-dose | Day 1 | 102438 | 101603 | 99644 | 94980 | 40577111 | 427.22 | 54.02% | 98.83% | 95.83% |
| A.1061 | Medium-dose | Day 1 | 106704 | 105946 | 104018 | 101030 | 43338552 | 428.97 | 54.35% | 98.94% | 96.13% |
| A.1062 | Medium-dose | Day 1 | 102480 | 101526 | 99701 | 97273 | 41721381 | 428.91 | 54.60% | 98.87% | 95.92% |
| A.1071 | Positive control | Day 1 | 106549 | 105664 | 103372 | 98204 | 42071932 | 428.41 | 54.24% | 98.74% | 95.52% |
| A.1072 | Positive control | Day 1 | 102183 | 101425 | 99459 | 96177 | 41191048 | 428.28 | 53.75% | 98.84% | 95.86% |
| A.1081 | Positive control | Day 1 | 68646 | 68046 | 66952 | 65127 | 27933743 | 428.91 | 54.12% | 98.99% | 96.29% |
| A.1082 | Positive control | Day 1 | 103075 | 102242 | 100732 | 99736 | 42784630 | 428.98 | 54.81% | 99.02% | 96.31% |
| A.1091 | Positive control | Day 1 | 109325 | 108483 | 106601 | 102667 | 44013228 | 428.70 | 53.87% | 98.85% | 95.89% |
| A.1092 | Positive control | Day 1 | 97973 | 97177 | 95324 | 92707 | 39756683 | 428.84 | 54.25% | 98.78% | 95.79% |
| A.1101 | High-dose | Day 1 | 63302 | 62835 | 61594 | 59951 | 25713014 | 428.90 | 53.34% | 98.85% | 95.92% |
| A.1102 | High-dose | Day 1 | 105871 | 105163 | 103356 | 101101 | 43352641 | 428.81 | 54.32% | 98.93% | 96.12% |
| A.1111 | High-dose | Day 1 | 105953 | 105032 | 103396 | 101599 | 43548315 | 428.63 | 54.19% | 98.89% | 96.01% |
| A.1112 | High-dose | Day 1 | 103434 | 102461 | 100695 | 99519 | 42582098 | 427.88 | 53.71% | 98.89% | 95.97% |
| A.1121 | High-dose | Day 1 | 107818 | 106865 | 104951 | 102431 | 43939928 | 428.97 | 53.77% | 98.84% | 95.89% |
| A.1122 | High-dose | Day 1 | 105485 | 104443 | 102542 | 99799 | 42814465 | 429.01 | 53.58% | 98.80% | 95.77% |
| A.1131 | Enrofloxacin | Day 1 | 80003 | 78952 | 77407 | 76398 | 32732474 | 428.45 | 54.44% | 98.28% | 94.21% |
| A.1132 | Enrofloxacin | Day 1 | 103082 | 102042 | 99745 | 99153 | 42531698 | 428.95 | 52.51% | 98.22% | 94.16% |
| A.1141 | Enrofloxacin | Day 1 | 105601 | 104187 | 101645 | 101151 | 43367515 | 428.74 | 52.63% | 97.98% | 93.57% |
| A.1142 | Enrofloxacin | Day 1 | 103173 | 101839 | 99910 | 98308 | 42108417 | 428.33 | 53.69% | 98.26% | 94.26% |
| A.1151 | Enrofloxacin | Day 1 | 105736 | 104486 | 102298 | 101223 | 43413033 | 428.89 | 54.73% | 98.39% | 94.48% |
| A.1152 | Enrofloxacin | Day 1 | 102482 | 100983 | 98665 | 97721 | 41881450 | 428.58 | 52.54% | 98.14% | 93.93% |
| A.1161 | Negative control | Day 1 | 102742 | 101594 | 99306 | 97248 | 41706618 | 428.87 | 53.45% | 98.21% | 94.13% |
| A.1162 | Negative control | Day 1 | 119708 | 118647 | 116141 | 113841 | 48811513 | 428.77 | 54.47% | 98.31% | 94.34% |
| A.1171 | Negative control | Day 1 | 110098 | 108929 | 106545 | 104585 | 44811615 | 428.47 | 53.64% | 98.28% | 94.27% |
| A.1172 | Negative control | Day 1 | 90492 | 89459 | 87571 | 86833 | 37217701 | 428.61 | 52.77% | 98.20% | 94.14% |
| A.1181 | Negative control | Day 1 | 93131 | 92331 | 90121 | 89517 | 38398067 | 428.95 | 52.52% | 98.21% | 94.19% |
| A.1182 | Negative control | Day 1 | 75839 | 75209 | 73560 | 73003 | 31284837 | 428.54 | 52.79% | 98.24% | 94.27% |
| B.2011 | Low-dose | Day 7 | 106541 | 105629 | 103309 | 99553 | 42716508 | 429.08 | 54.48% | 98.40% | 94.52% |
| B.2012 | Low-dose | Day 7 | 102691 | 101688 | 99520 | 96670 | 41473001 | 429.02 | 54.35% | 98.36% | 94.43% |
| B.2021 | Low-dose | Day 7 | 107514 | 106488 | 104392 | 102933 | 44154396 | 428.96 | 54.89% | 98.46% | 94.73% |
| B.2022 | Low-dose | Day 7 | 106319 | 105201 | 103002 | 99205 | 42431789 | 427.72 | 54.19% | 98.26% | 94.19% |
| B.2031 | Low-dose | Day 7 | 154164 | 153432 | 150637 | 146115 | 62688385 | 429.03 | 54.83% | 98.63% | 95.20% |
| B.2032 | Low-dose | Day 7 | 105590 | 104621 | 102441 | 99731 | 42786851 | 429.02 | 54.41% | 98.43% | 94.69% |
| B.2041 | Medium-dose | Day 7 | 116975 | 115586 | 113170 | 108587 | 46572837 | 428.90 | 54.59% | 98.34% | 94.36% |
| B.2042 | Medium-dose | Day 7 | 105392 | 104110 | 102135 | 101137 | 43336737 | 428.50 | 54.93% | 98.41% | 94.44% |
| B.2051 | Medium-dose | Day 7 | 102925 | 101864 | 99983 | 95848 | 41110789 | 428.92 | 55.04% | 98.52% | 94.90% |
| B.2052 | Medium-dose | Day 7 | 104615 | 103269 | 101235 | 94413 | 40470519 | 428.65 | 55.30% | 98.29% | 94.22% |
| B.2061 | Medium-dose | Day 7 | 103046 | 102077 | 99946 | 97093 | 41644524 | 428.91 | 54.89% | 98.34% | 94.40% |
| B.2062 | Medium-dose | Day 7 | 106247 | 105213 | 103099 | 102356 | 43823765 | 428.15 | 54.86% | 98.34% | 94.36% |
| B.2071 | Positive control | Day 7 | 106727 | 105749 | 103459 | 97987 | 41953496 | 428.15 | 55.43% | 98.45% | 94.70% |
| B.2072 | Positive control | Day 7 | 106307 | 105193 | 102736 | 99598 | 42691431 | 428.64 | 55.18% | 98.12% | 93.84% |
| B.2081 | Positive control | Day 7 | 112570 | 111282 | 108973 | 103556 | 44424103 | 428.99 | 55.83% | 98.38% | 94.54% |
| B.2082 | Positive control | Day 7 | 103435 | 102458 | 100142 | 96551 | 41399965 | 428.79 | 55.02% | 98.38% | 94.51% |
| B.2091 | Positive control | Day 7 | 102652 | 101682 | 99506 | 93243 | 39968640 | 428.65 | 55.37% | 98.36% | 94.41% |
| B.2092 | Positive control | Day 7 | 105493 | 104503 | 102158 | 92897 | 39740744 | 427.79 | 54.83% | 98.30% | 94.34% |
| B.2101 | High-dose | Day 7 | 105866 | 105009 | 102900 | 98552 | 42228759 | 428.49 | 55.51% | 98.35% | 94.35% |
| B.2102 | High-dose | Day 7 | 115441 | 113646 | 111199 | 105510 | 45259162 | 428.96 | 55.35% | 98.23% | 94.09% |
| B.2111 | High-dose | Day 7 | 112317 | 111180 | 108941 | 102483 | 43962632 | 428.97 | 54.79% | 98.42% | 94.55% |
| B.2112 | High-dose | Day 7 | 104124 | 102916 | 100716 | 96167 | 41240676 | 428.84 | 55.23% | 98.42% | 94.55% |
| B.2121 | High-dose | Day 7 | 113994 | 112975 | 110433 | 102775 | 44089848 | 428.99 | 54.99% | 98.45% | 94.67% |
| B.2122 | High-dose | Day 7 | 106531 | 105654 | 103455 | 99596 | 42723652 | 428.97 | 55.76% | 98.48% | 94.76% |
| B.2131 | Enrofloxacin | Day 7 | 105687 | 104826 | 102454 | 101400 | 43489591 | 428.89 | 54.66% | 98.42% | 94.64% |
| B.2132 | Enrofloxacin | Day 7 | 70591 | 70066 | 68773 | 67747 | 29063278 | 429.00 | 54.72% | 98.52% | 94.93% |
| B.2141 | Enrofloxacin | Day 7 | 159159 | 158223 | 155386 | 148006 | 62971529 | 425.47 | 54.51% | 98.50% | 94.75% |
| B.2142 | Enrofloxacin | Day 7 | 106439 | 105143 | 102904 | 101354 | 43476507 | 428.96 | 54.69% | 98.39% | 94.46% |
| B.2151 | Enrofloxacin | Day 7 | 105458 | 104409 | 102077 | 100520 | 43121643 | 428.99 | 54.80% | 98.41% | 94.62% |
| B.2152 | Enrofloxacin | Day 7 | 57291 | 56813 | 55627 | 55222 | 23689208 | 428.98 | 54.90% | 98.39% | 94.61% |
| B.2161 | Negative control | Day 7 | 102413 | 101452 | 99312 | 96096 | 41218551 | 428.93 | 54.87% | 98.33% | 94.29% |
| B.2162 | Negative control | Day 7 | 103417 | 102228 | 100267 | 97516 | 41831284 | 428.97 | 55.15% | 98.43% | 94.61% |
| B.2171 | Negative control | Day 7 | 54619 | 54036 | 53023 | 50916 | 21841683 | 428.97 | 54.96% | 98.26% | 94.22% |
| B.2172 | Negative control | Day 7 | 105316 | 104352 | 102445 | 98125 | 42092425 | 428.97 | 55.18% | 98.59% | 95.02% |
| B.2181 | Negative control | Day 7 | 82720 | 81777 | 80067 | 75583 | 32421090 | 428.95 | 55.31% | 98.31% | 94.33% |
| B.2182 | Negative control | Day 7 | 113919 | 112721 | 110453 | 107702 | 46199491 | 428.96 | 54.84% | 98.40% | 94.47% |
| C.3011 | Low-dose | Day 42 | 101608 | 101056 | 99781 | 92334 | 39555338 | 428.39 | 54.61% | 98.92% | 96.05% |
| C.3012 | Low-dose | Day 42 | 102694 | 101896 | 100536 | 88563 | 37864982 | 427.55 | 54.00% | 98.83% | 95.80% |
| C.3021 | Low-dose | Day 42 | 102943 | 102089 | 100522 | 93612 | 40065100 | 427.99 | 51.71% | 98.77% | 95.47% |
| C.3022 | Low-dose | Day 42 | 106100 | 105426 | 104098 | 96121 | 41107646 | 427.67 | 52.20% | 98.96% | 96.10% |
| C.3031 | Low-dose | Day 42 | 103939 | 103066 | 101551 | 93625 | 39982737 | 427.05 | 52.89% | 98.84% | 95.71% |
| C.3032 | Low-dose | Day 42 | 106239 | 105433 | 104023 | 94868 | 40565917 | 427.60 | 53.28% | 98.88% | 95.85% |
| C.3041 | Medium-dose | Day 42 | 105001 | 104205 | 102735 | 95369 | 40571780 | 425.42 | 51.78% | 98.62% | 95.15% |
| C.3042 | Medium-dose | Day 42 | 117487 | 116644 | 115115 | 104042 | 44213296 | 424.96 | 50.86% | 98.83% | 95.74% |
| C.3051 | Medium-dose | Day 42 | 108587 | 107853 | 106311 | 95493 | 40757987 | 426.82 | 52.16% | 98.80% | 95.66% |
| C.3052 | Medium-dose | Day 42 | 117310 | 116485 | 114795 | 94647 | 40325019 | 426.06 | 51.72% | 98.83% | 95.73% |
| C.3061 | Medium-dose | Day 42 | 105920 | 105172 | 103518 | 79178 | 33704106 | 425.68 | 51.46% | 98.67% | 95.31% |
| C.3062 | Medium-dose | Day 42 | 119968 | 119127 | 117367 | 96832 | 41219893 | 425.68 | 51.01% | 98.82% | 95.73% |
| C.3071 | Positive control | Day 42 | 102030 | 101335 | 100186 | 95632 | 40880489 | 427.48 | 53.41% | 99.03% | 96.31% |
| C.3072 | Positive control | Day 42 | 102270 | 101626 | 100269 | 93906 | 40128278 | 427.32 | 53.20% | 98.93% | 96.05% |
| C.3081 | Positive control | Day 42 | 102073 | 101305 | 99865 | 90327 | 38391322 | 425.03 | 51.12% | 98.86% | 95.66% |
| C.3082 | Positive control | Day 42 | 105974 | 105182 | 103699 | 90395 | 38409508 | 424.91 | 51.35% | 98.87% | 95.76% |
| C.3091 | Positive control | Day 42 | 102141 | 101609 | 100428 | 97428 | 41543011 | 426.40 | 52.84% | 98.97% | 96.20% |
| C.3092 | Positive control | Day 42 | 103768 | 103039 | 101511 | 91376 | 38951385 | 426.28 | 51.43% | 98.75% | 95.40% |
| C.3101 | High-dose | Day 42 | 102706 | 102011 | 100524 | 95387 | 40631420 | 425.96 | 53.89% | 98.87% | 95.89% |
| C.3102 | High-dose | Day 42 | 112113 | 111319 | 109663 | 99010 | 42272585 | 426.95 | 51.49% | 98.82% | 95.69% |
| C.3111 | High-dose | Day 42 | 103809 | 103154 | 101666 | 90594 | 38662531 | 426.77 | 52.68% | 98.83% | 95.77% |
| C.3112 | High-dose | Day 42 | 106693 | 105972 | 104482 | 63088 | 26922382 | 426.74 | 52.43% | 98.81% | 95.62% |
| C.3121 | High-dose | Day 42 | 119817 | 118878 | 117031 | 72606 | 31113625 | 428.53 | 52.55% | 98.88% | 95.79% |
| C.3122 | High-dose | Day 42 | 112674 | 111479 | 109699 | 71962 | 30794500 | 427.93 | 51.55% | 98.79% | 95.54% |
| C.3131 | Enrofloxacin | Day 42 | 104653 | 104036 | 102574 | 76748 | 32723023 | 426.37 | 53.22% | 98.94% | 96.05% |
| C.3132 | Enrofloxacin | Day 42 | 104030 | 103207 | 101557 | 65460 | 27863096 | 425.65 | 53.03% | 98.78% | 95.66% |
| C.3141 | Enrofloxacin | Day 42 | 103156 | 102518 | 101231 | 62309 | 26485415 | 425.07 | 50.77% | 98.89% | 95.92% |
| C.3142 | Enrofloxacin | Day 42 | 96383 | 95815 | 94252 | 56751 | 24077283 | 424.26 | 53.63% | 98.72% | 95.48% |
| C.3151 | Enrofloxacin | Day 42 | 104001 | 103091 | 101527 | 59605 | 25467464 | 427.27 | 52.57% | 98.85% | 95.73% |
| C.3152 | Enrofloxacin | Day 42 | 104202 | 103436 | 101733 | 58327 | 24939469 | 427.58 | 52.72% | 98.78% | 95.70% |
| C.3162 | Negative control | Day 42 | 104624 | 103701 | 101378 | 84256 | 36059633 | 427.98 | 52.29% | 98.04% | 93.67% |
| C.3171 | Negative control | Day 42 | 106238 | 105239 | 103384 | 80949 | 34720206 | 428.91 | 52.97% | 98.39% | 94.35% |
| C.3172 | Negative control | Day 42 | 103821 | 103142 | 101614 | 95005 | 40696125 | 428.36 | 54.61% | 98.88% | 95.84% |
| C.3181 | Negative control | Day 42 | 104703 | 103824 | 102503 | 57417 | 24436908 | 425.60 | 52.53% | 98.98% | 96.16% |
| C.3182 | Negative control | Day 42 | 104109 | 103296 | 101556 | 66460 | 28417920 | 427.59 | 53.23% | 98.49% | 94.74% |


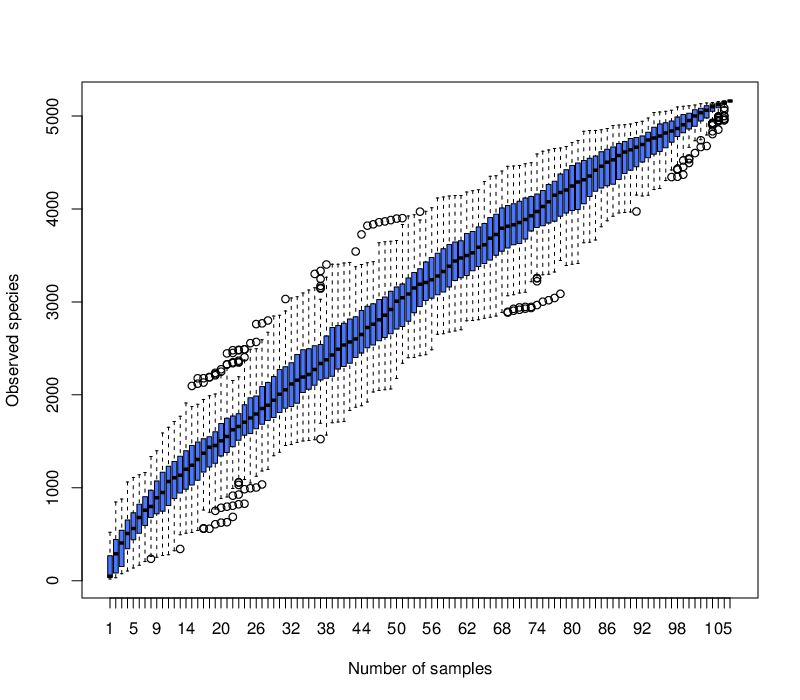


**Supplementary Figure 1** Species accumulation boxplot of intestinal microbiota samples


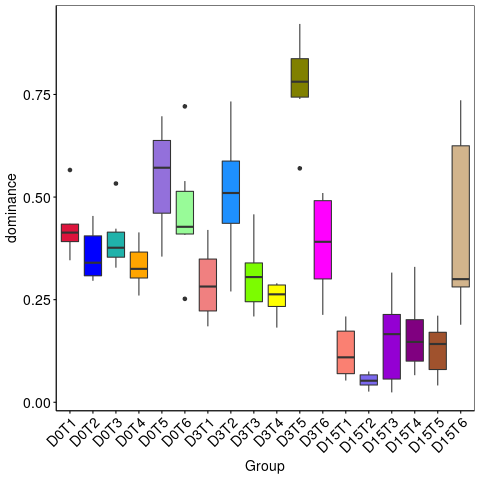


**Supplementary Figure 2** Dominance index of microbial communities across groups and time points at day 1 (D0T), day 7 (D3T), and day 42 (D15). 1 – low-dose, 2 – medium-dose, 3 – positive control, 4 – high-dose, 5 – enrofloxacin, 6 – negative control.


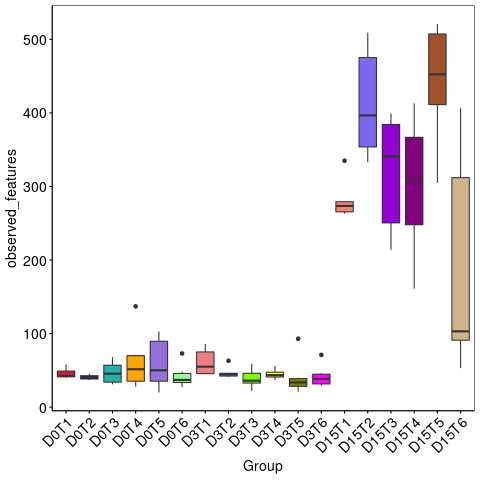


**Supplementary Figure 3** Number of observed features (richness) across groups and time points at day 1 (D0T), day 7 (D3T), and day 42 (D15). 1 – low-dose, 2 – medium-dose, 3 – positive control, 4 – high-dose, 5 – enrofloxacin, 6 – negative control.


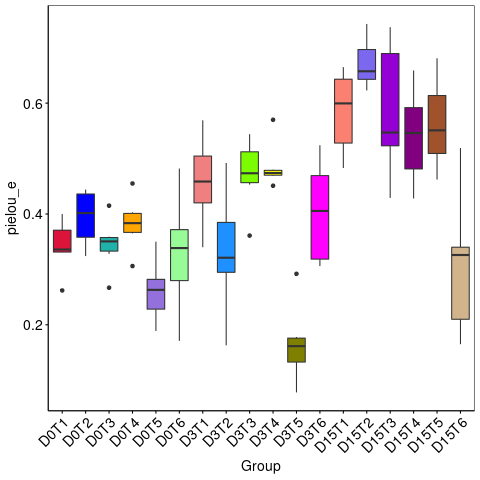


**Supplementary Figure 4** Pielou’s evenness index across groups and time points at day 1 (D0T), day 7 (D3T), and day 42 (D15). 1 – low-dose, 2 – medium-dose, 3 – positive control, 4 – high-dose, 5 – enrofloxacin, 6 – negative control.


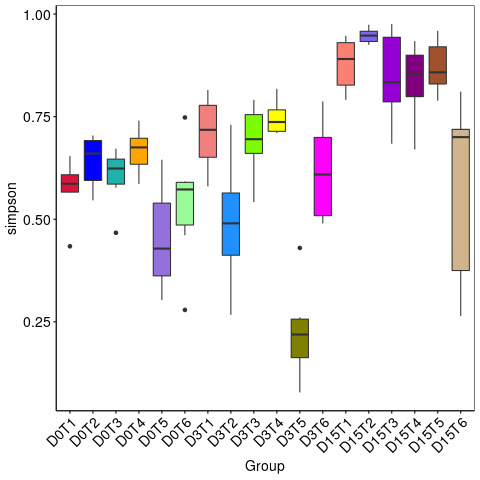


**Supplementary Figure 5** Simpson diversity index across groups and time points at day 1 (D0T), day 7 (D3T), and day 42 (D15). 1 – low-dose, 2 – medium-dose, 3 – positive control, 4 – high-dose, 5 – enrofloxacin, 6 – negative control.


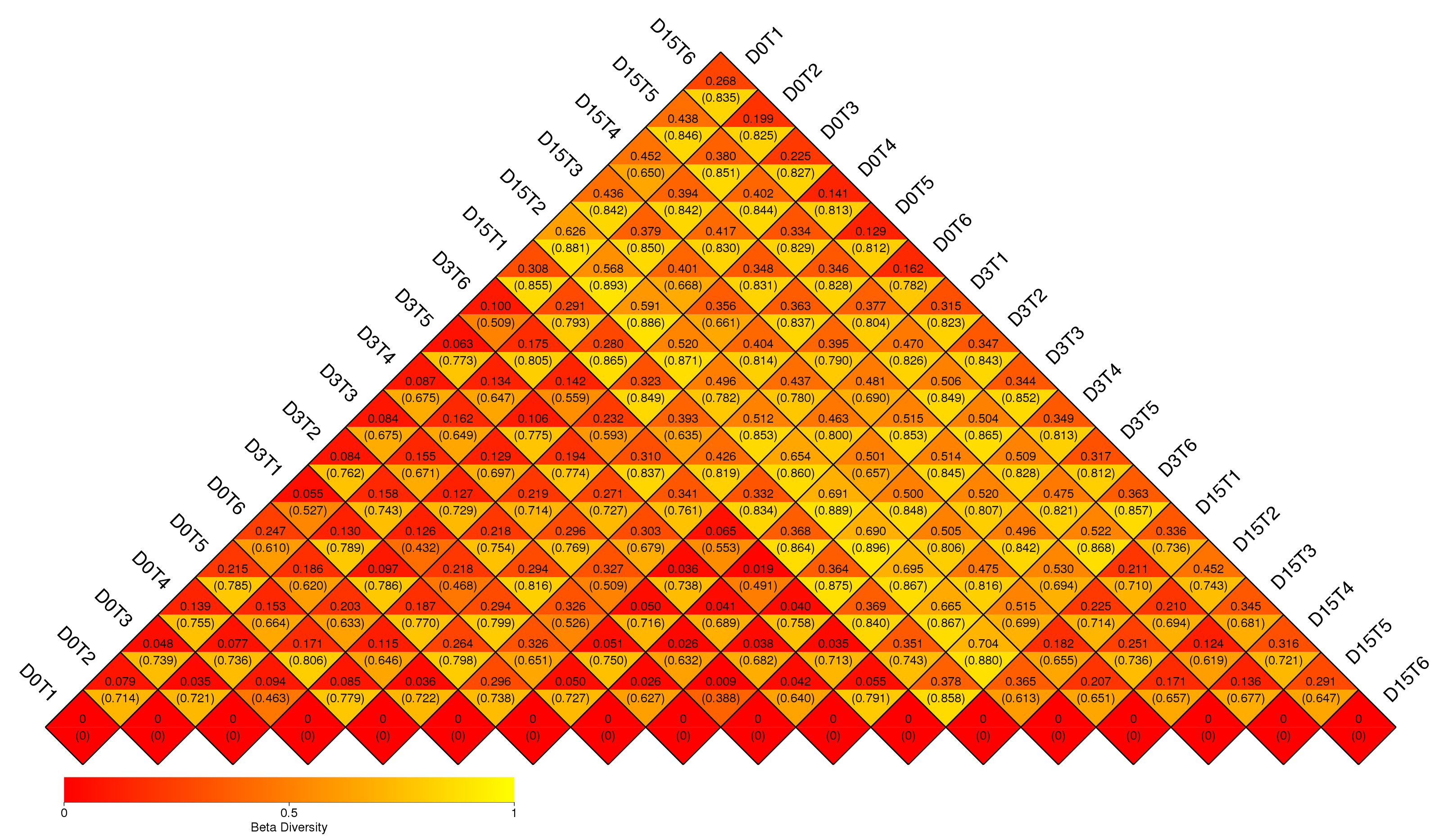


**Supplementary Figure 6** Heatmap of beta diversity based on Weighted UniFrac distances across groups and time points. Day 1 („D0T”), day 7 (D3T), and day 42 (D15). 1 – low-dose, 2 – medium-dose, 3 – positive control, 4 – high-dose, 5 – enrofloxacin, 6 – negative control.


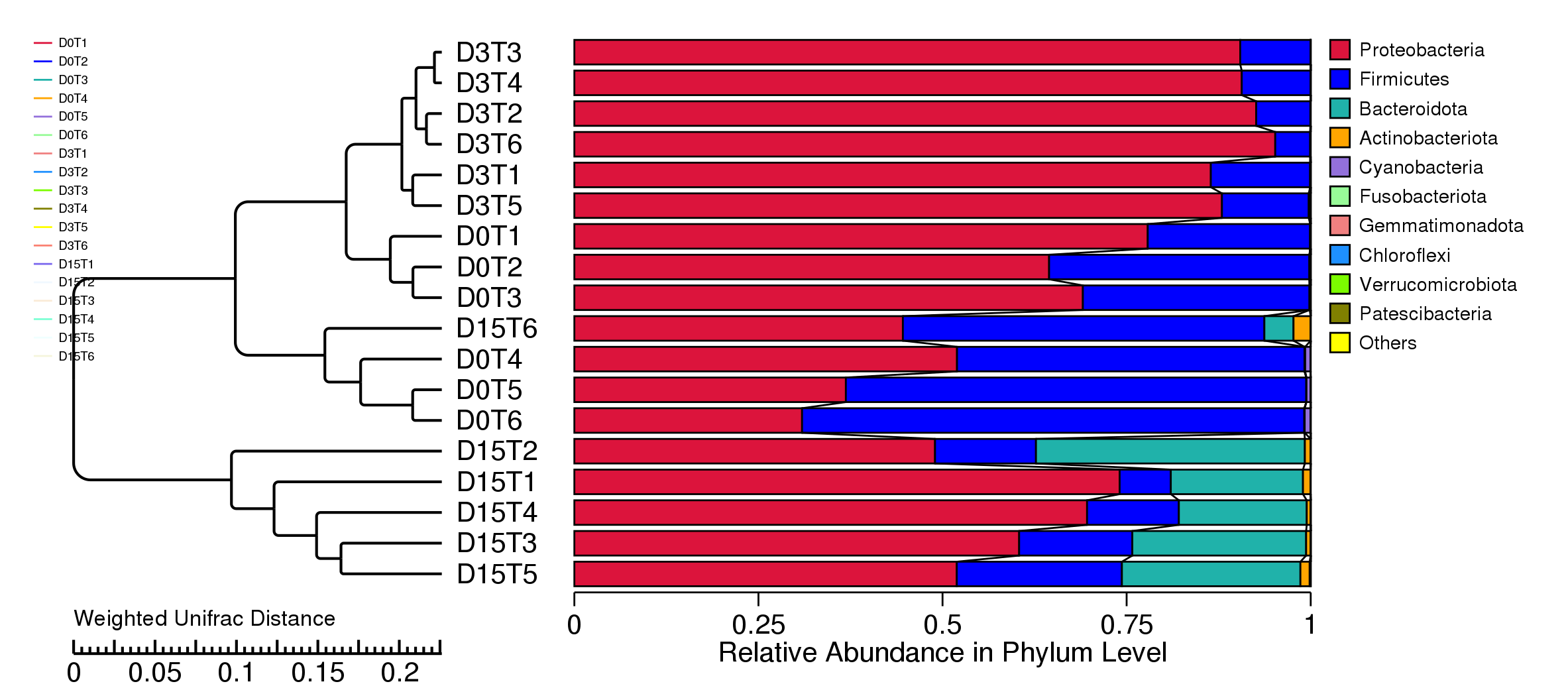


**Supplementary Figure 7** Unweighted Pair-group Method with Arithmetic Mean (UPGMA) dendrogram and phylum-level relative abundance. Hierarchical clustering (based on Weighted UniFrac distance) and relative abundance of major taxa in the studied groups at day 1 (D0T), day 7 (D3T), and day 42 (D15). 1 – low-dose, 2 – medium-dose, 3 – positive control, 4 – high-dose, 5 – enrofloxacin, 6 – negative control.

**Supplementary Table 6** Alpha diversity indices of the intestinal microbiota across treatment groups and time points

| **Sample** | **Group** | **Day** | **chao1** | **Dominance** | **Goods coverage** | **Observed features** | **Pielou_e** | **Shannon** | **Simpson** |
| --- | --- | --- | --- | --- | --- | --- | --- | --- | --- |
| A.1011 | Low-dose | Day 1 | 42 | 0.431 | 1 | 42 | 0.331 | 1.783 | 0.569 |
| A.1012 | Low-dose | Day 1 | 51.6 | 0.566 | 1 | 51 | 0.262 | 1.485 | 0.434 |
| A.1021 | Low-dose | Day 1 | 45.5 | 0.346 | 1 | 43 | 0.4 | 2.171 | 0.654 |
| A.1022 | Low-dose | Day 1 | 45.2 | 0.435 | 1 | 41 | 0.332 | 1.78 | 0.565 |
| A.1031 | Low-dose | Day 1 | 42 | 0.39 | 1 | 40 | 0.381 | 2.027 | 0.61 |
| A.1032 | Low-dose | Day 1 | 58.909 | 0.396 | 1 | 58 | 0.34 | 1.992 | 0.604 |
| A.1041 | Medium-dose | Day 1 | 44.333 | 0.368 | 1 | 42 | 0.385 | 2.077 | 0.632 |
| A.1042 | Medium-dose | Day 1 | 49.5 | 0.312 | 1 | 45 | 0.444 | 2.436 | 0.688 |
| A.1051 | Medium-dose | Day 1 | 49 | 0.307 | 1 | 43 | 0.418 | 2.268 | 0.693 |
| A.1052 | Medium-dose | Day 1 | 41.5 | 0.296 | 1 | 39 | 0.442 | 2.335 | 0.704 |
| A.1061 | Medium-dose | Day 1 | 40.5 | 0.418 | 1 | 38 | 0.349 | 1.832 | 0.582 |
| A.1062 | Medium-dose | Day 1 | 40 | 0.454 | 1 | 37 | 0.324 | 1.689 | 0.546 |
| A.1071 | Positive control | Day 1 | 62.6 | 0.389 | 1 | 59 | 0.328 | 1.931 | 0.611 |
| A.1072 | Positive control | Day 1 | 34 | 0.328 | 1 | 31 | 0.415 | 2.057 | 0.672 |
| A.1081 | Positive control | Day 1 | 69.2 | 0.364 | 1 | 68 | 0.348 | 2.121 | 0.636 |
| A.1082 | Positive control | Day 1 | 34 | 0.533 | 1 | 32 | 0.267 | 1.334 | 0.467 |
| A.1091 | Positive control | Day 1 | 40.2 | 0.35 | 1 | 40 | 0.359 | 1.913 | 0.65 |
| A.1092 | Positive control | Day 1 | 52 | 0.423 | 1 | 51 | 0.353 | 2.001 | 0.577 |
| A.1101 | High-dose | Day 1 | 68.111 | 0.378 | 1 | 67 | 0.306 | 1.856 | 0.622 |
| A.1102 | High-dose | Day 1 | 31 | 0.414 | 1 | 28 | 0.372 | 1.79 | 0.586 |
| A.1111 | High-dose | Day 1 | 77.429 | 0.33 | 1 | 71 | 0.403 | 2.476 | 0.67 |
| A.1112 | High-dose | Day 1 | 140.438 | 0.297 | 1 | 137 | 0.365 | 2.592 | 0.703 |
| A.1121 | High-dose | Day 1 | 36.5 | 0.26 | 1 | 36 | 0.455 | 2.353 | 0.74 |
| A.1122 | High-dose | Day 1 | 35.375 | 0.32 | 1 | 35 | 0.395 | 2.026 | 0.68 |
| A.1131 | Enrofloxacin | Day 1 | 113.2 | 0.427 | 1 | 100 | 0.288 | 1.914 | 0.573 |
| A.1132 | Enrofloxacin | Day 1 | 20 | 0.697 | 1 | 20 | 0.217 | 0.937 | 0.303 |
| A.1141 | Enrofloxacin | Day 1 | 65.8 | 0.657 | 1 | 58 | 0.189 | 1.109 | 0.343 |
| A.1142 | Enrofloxacin | Day 1 | 53 | 0.355 | 1 | 42 | 0.35 | 1.885 | 0.645 |
| A.1151 | Enrofloxacin | Day 1 | 36.333 | 0.581 | 1 | 33 | 0.262 | 1.323 | 0.419 |
| A.1152 | Enrofloxacin | Day 1 | 106.056 | 0.562 | 1 | 103 | 0.264 | 1.766 | 0.438 |
| A.1161 | Negative control | Day 1 | 35 | 0.408 | 1 | 33 | 0.336 | 1.697 | 0.592 |
| A.1162 | Negative control | Day 1 | 28 | 0.416 | 1 | 27 | 0.382 | 1.817 | 0.584 |
| A.1171 | Negative control | Day 1 | 39.167 | 0.252 | 1 | 39 | 0.482 | 2.546 | 0.748 |
| A.1172 | Negative control | Day 1 | 51.6 | 0.539 | 1 | 48 | 0.261 | 1.458 | 0.461 |
| A.1181 | Negative control | Day 1 | 35.75 | 0.721 | 1 | 35 | 0.171 | 0.875 | 0.279 |
| A.1182 | Negative control | Day 1 | 73.25 | 0.439 | 1 | 73 | 0.341 | 2.111 | 0.561 |
| B.2011 | Low-dose | Day 7 | 65.143 | 0.31 | 1 | 63 | 0.409 | 2.445 | 0.69 |
| B.2012 | Low-dose | Day 7 | 87.667 | 0.254 | 1 | 86 | 0.453 | 2.913 | 0.746 |
| B.2021 | Low-dose | Day 7 | 46.2 | 0.42 | 1 | 45 | 0.34 | 1.867 | 0.58 |
| B.2022 | Low-dose | Day 7 | 82.5 | 0.185 | 1 | 79 | 0.518 | 3.267 | 0.815 |
| B.2031 | Low-dose | Day 7 | 47.5 | 0.212 | 1 | 47 | 0.569 | 3.161 | 0.788 |
| B.2032 | Low-dose | Day 7 | 46 | 0.362 | 1 | 45 | 0.464 | 2.55 | 0.638 |
| B.2041 | Medium-dose | Day 7 | 65.154 | 0.469 | 1 | 63 | 0.33 | 1.974 | 0.531 |
| B.2042 | Medium-dose | Day 7 | 61 | 0.733 | 1 | 46 | 0.163 | 0.899 | 0.267 |
| B.2051 | Medium-dose | Day 7 | 41.75 | 0.425 | 1 | 41 | 0.403 | 2.157 | 0.575 |
| B.2052 | Medium-dose | Day 7 | 43 | 0.27 | 1 | 42 | 0.492 | 2.651 | 0.73 |
| B.2061 | Medium-dose | Day 7 | 43.75 | 0.6 | 1 | 43 | 0.289 | 1.567 | 0.4 |
| B.2062 | Medium-dose | Day 7 | 51 | 0.551 | 1 | 46 | 0.312 | 1.725 | 0.449 |
| B.2071 | Positive control | Day 7 | 32 | 0.317 | 1 | 32 | 0.453 | 2.264 | 0.683 |
| B.2072 | Positive control | Day 7 | 50 | 0.458 | 1 | 49 | 0.361 | 2.029 | 0.542 |
| B.2081 | Positive control | Day 7 | 22 | 0.229 | 1 | 22 | 0.544 | 2.424 | 0.771 |
| B.2082 | Positive control | Day 7 | 38.5 | 0.347 | 1 | 37 | 0.467 | 2.433 | 0.653 |
| B.2091 | Positive control | Day 7 | 35 | 0.293 | 1 | 35 | 0.48 | 2.463 | 0.707 |
| B.2092 | Positive control | Day 7 | 60 | 0.209 | 1 | 59 | 0.523 | 3.074 | 0.791 |
| B.2101 | High-dose | Day 7 | 59 | 0.241 | 1 | 56 | 0.469 | 2.724 | 0.759 |
| B.2102 | High-dose | Day 7 | 42 | 0.285 | 1 | 41 | 0.473 | 2.532 | 0.715 |
| B.2111 | High-dose | Day 7 | 43.5 | 0.182 | 1 | 41 | 0.57 | 3.055 | 0.818 |
| B.2112 | High-dose | Day 7 | 46.6 | 0.29 | 1 | 46 | 0.451 | 2.489 | 0.71 |
| B.2121 | High-dose | Day 7 | 55.2 | 0.286 | 1 | 48 | 0.48 | 2.682 | 0.714 |
| B.2122 | High-dose | Day 7 | 40.75 | 0.231 | 1 | 37 | 0.475 | 2.476 | 0.769 |
| B.2131 | Enrofloxacin | Day 7 | 43 | 0.739 | 1 | 40 | 0.17 | 0.903 | 0.261 |
| B.2132 | Enrofloxacin | Day 7 | 28 | 0.805 | 1 | 27 | 0.153 | 0.729 | 0.195 |
| B.2141 | Enrofloxacin | Day 7 | 96.75 | 0.57 | 1 | 93 | 0.292 | 1.909 | 0.43 |
| B.2142 | Enrofloxacin | Day 7 | 38 | 0.757 | 1 | 35 | 0.178 | 0.912 | 0.243 |
| B.2151 | Enrofloxacin | Day 7 | 33.5 | 0.848 | 1 | 32 | 0.126 | 0.628 | 0.152 |
| B.2152 | Enrofloxacin | Day 7 | 21 | 0.922 | 1 | 21 | 0.078 | 0.344 | 0.078 |
| B.2161 | Negative control | Day 7 | 78.583 | 0.48 | 1 | 71 | 0.309 | 1.898 | 0.52 |
| B.2162 | Negative control | Day 7 | 32 | 0.495 | 1 | 29 | 0.347 | 1.688 | 0.505 |
| B.2171 | Negative control | Day 7 | 30 | 0.3 | 1 | 30 | 0.471 | 2.311 | 0.7 |
| B.2172 | Negative control | Day 7 | 45.2 | 0.302 | 1 | 41 | 0.464 | 2.486 | 0.698 |
| B.2181 | Negative control | Day 7 | 36.333 | 0.213 | 1 | 36 | 0.524 | 2.71 | 0.787 |
| B.2182 | Negative control | Day 7 | 47.5 | 0.51 | 1 | 46 | 0.306 | 1.687 | 0.49 |
| C.3011 | Low-dose | Day 42 | 277.034 | 0.184 | 0.999 | 264 | 0.483 | 3.882 | 0.816 |
| C.3012 | Low-dose | Day 42 | 283.955 | 0.141 | 1 | 277 | 0.564 | 4.576 | 0.859 |
| C.3021 | Low-dose | Day 42 | 271.75 | 0.053 | 1 | 263 | 0.665 | 5.349 | 0.947 |
| C.3022 | Low-dose | Day 42 | 288.143 | 0.067 | 1 | 280 | 0.635 | 5.162 | 0.933 |
| C.3031 | Low-dose | Day 42 | 344.625 | 0.078 | 1 | 335 | 0.646 | 5.421 | 0.922 |
| C.3032 | Low-dose | Day 42 | 278 | 0.209 | 1 | 270 | 0.516 | 4.164 | 0.791 |
| C.3041 | Medium-dose | Day 42 | 380 | 0.075 | 0.999 | 371 | 0.623 | 5.321 | 0.925 |
| C.3042 | Medium-dose | Day 42 | 357.2 | 0.051 | 0.999 | 348 | 0.655 | 5.533 | 0.949 |
| C.3051 | Medium-dose | Day 42 | 346.097 | 0.071 | 0.999 | 333 | 0.639 | 5.356 | 0.929 |
| C.3052 | Medium-dose | Day 42 | 474.059 | 0.054 | 0.999 | 422 | 0.66 | 5.755 | 0.946 |
| C.3061 | Medium-dose | Day 42 | 528.528 | 0.039 | 0.999 | 509 | 0.709 | 6.372 | 0.961 |
| C.3062 | Medium-dose | Day 42 | 512.5 | 0.026 | 0.999 | 493 | 0.743 | 6.647 | 0.974 |
| C.3071 | Positive control | Day 42 | 236.25 | 0.224 | 1 | 231 | 0.519 | 4.073 | 0.776 |
| C.3072 | Positive control | Day 42 | 220.5 | 0.184 | 1 | 214 | 0.535 | 4.142 | 0.816 |
| C.3081 | Positive control | Day 42 | 375.935 | 0.026 | 1 | 373 | 0.733 | 6.264 | 0.974 |
| C.3082 | Positive control | Day 42 | 410.5 | 0.024 | 0.999 | 388 | 0.737 | 6.338 | 0.976 |
| C.3091 | Positive control | Day 42 | 409.029 | 0.316 | 0.999 | 399 | 0.429 | 3.709 | 0.684 |
| C.3092 | Positive control | Day 42 | 313 | 0.148 | 1 | 309 | 0.559 | 4.626 | 0.852 |
| C.3101 | High-dose | Day 42 | 166.571 | 0.33 | 1 | 161 | 0.428 | 3.137 | 0.67 |
| C.3102 | High-dose | Day 42 | 256.462 | 0.066 | 1 | 246 | 0.659 | 5.231 | 0.934 |
| C.3111 | High-dose | Day 42 | 260.375 | 0.119 | 1 | 254 | 0.597 | 4.767 | 0.881 |
| C.3112 | High-dose | Day 42 | 380.467 | 0.094 | 0.999 | 368 | 0.577 | 4.918 | 0.906 |
| C.3121 | High-dose | Day 42 | 381.571 | 0.21 | 0.999 | 363 | 0.47 | 4 | 0.79 |
| C.3122 | High-dose | Day 42 | 435.061 | 0.175 | 0.999 | 413 | 0.515 | 4.478 | 0.825 |
| C.3131 | Enrofloxacin | Day 42 | 310.163 | 0.211 | 1 | 305 | 0.462 | 3.812 | 0.789 |
| C.3132 | Enrofloxacin | Day 42 | 409.382 | 0.178 | 0.999 | 402 | 0.497 | 4.302 | 0.822 |
| C.3141 | Enrofloxacin | Day 42 | 445.268 | 0.041 | 0.999 | 439 | 0.681 | 5.978 | 0.959 |
| C.3142 | Enrofloxacin | Day 42 | 477.071 | 0.061 | 0.999 | 466 | 0.633 | 5.613 | 0.939 |
| C.3151 | Enrofloxacin | Day 42 | 531.652 | 0.148 | 0.999 | 521 | 0.556 | 5.015 | 0.852 |
| C.3152 | Enrofloxacin | Day 42 | 531.586 | 0.136 | 0.999 | 521 | 0.546 | 4.932 | 0.864 |
| C.3162 | Negative control | Day 42 | 109 | 0.625 | 1 | 103 | 0.21 | 1.403 | 0.375 |
| C.3171 | Negative control | Day 42 | 94.462 | 0.3 | 1 | 91 | 0.326 | 2.122 | 0.7 |
| C.3172 | Negative control | Day 42 | 58 | 0.736 | 1 | 53 | 0.165 | 0.945 | 0.264 |
| C.3181 | Negative control | Day 42 | 409.065 | 0.189 | 1 | 406 | 0.519 | 4.5 | 0.811 |
| C.3182 | Negative control | Day 42 | 322.783 | 0.281 | 0.999 | 312 | 0.34 | 2.815 | 0.719 |

**Supplementary Table S7.** Overall group comparisons for alpha-diversity indices at each sampling time point. Alpha diversity was calculated in QIIME2 and evaluated for Shannon diversity, Chao1 richness, Observed features (ASVs), Simpson index, Dominance, Pielou’s evenness, and Good’s coverage. For each time point (Day 1, Day 7, and Day 42), group-level differences among treatments (T1–T6) were tested using the Kruskal–Wallis test (df = 5). Sample sizes are reported for each treatment and time point; Day 42 includes one missing sample in the negative control (T6). For Good’s coverage, statistical testing is not applicable (NA) when values are invariant across groups. Treatment key: T1 low-dose phytobiotic, T2 medium-dose phytobiotic, T3 positive control (infected, untreated), T4 high-dose phytobiotic, T5 enrofloxacin, T6 negative control (uninfected, untreated).

| Time point | Metric | n (T1) | n (T2) | n (T3) | n (T4) | n (T5) | n (T6) | Kruskal–Wallis H | df | p-value |
| --- | --- | --- | --- | --- | --- | --- | --- | --- | --- | --- |
| Day 1 | Shannon diversity | 6 | 6 | 6 | 6 | 6 | 6 | 9.712 | 5 | 0.083828 |
| Day 1 | Chao1 richness | 6 | 6 | 6 | 6 | 6 | 6 | 2.469 | 5 | 0.781206 |
| Day 1 | Observed features (ASVs) | 6 | 6 | 6 | 6 | 6 | 6 | 1.677 | 5 | 0.891747 |
| Day 1 | Simpson index | 6 | 6 | 6 | 6 | 6 | 6 | 12.474 | 5 | 0.028834 |
| Day 1 | Dominance | 6 | 6 | 6 | 6 | 6 | 6 | 12.474 | 5 | 0.028834 |
| Day 1 | Pielou's evenness | 6 | 6 | 6 | 6 | 6 | 6 | 11.899 | 5 | 0.036203 |
| Day 1 | Good's coverage | 6 | 6 | 6 | 6 | 6 | 6 | NA | 5 | NA |
| Day 7 | Shannon diversity | 6 | 6 | 6 | 6 | 6 | 6 | 20.538 | 5 | 0.00099 |
| Day 7 | Chao1 richness | 6 | 6 | 6 | 6 | 6 | 6 | 9.308 | 5 | 0.097378 |
| Day 7 | Observed features (ASVs) | 6 | 6 | 6 | 6 | 6 | 6 | 9.885 | 5 | 0.078548 |
| Day 7 | Simpson index | 6 | 6 | 6 | 6 | 6 | 6 | 21.949 | 5 | 0.000535 |
| Day 7 | Dominance | 6 | 6 | 6 | 6 | 6 | 6 | 21.949 | 5 | 0.000535 |
| Day 7 | Pielou's evenness | 6 | 6 | 6 | 6 | 6 | 6 | 19.63 | 5 | 0.001466 |
| Day 7 | Good's coverage | 6 | 6 | 6 | 6 | 6 | 6 | NA | 5 | NA |
| Day 42 | Shannon diversity | 6 | 6 | 6 | 6 | 6 | 5 | 16.516 | 5 | 0.005516 |
| Day 42 | Chao1 richness | 6 | 6 | 6 | 6 | 6 | 5 | 15.298 | 5 | 0.009162 |
| Day 42 | Observed features (ASVs) | 6 | 6 | 6 | 6 | 6 | 5 | 15.117 | 5 | 0.009875 |
| Day 42 | Simpson index | 6 | 6 | 6 | 6 | 6 | 5 | 14.77 | 5 | 0.011393 |
| Day 42 | Dominance | 6 | 6 | 6 | 6 | 6 | 5 | 14.77 | 5 | 0.011393 |
| Day 42 | Pielou's evenness | 6 | 6 | 6 | 6 | 6 | 5 | 15.348 | 5 | 0.008974 |
| Day 42 | Good's coverage | 6 | 6 | 6 | 6 | 6 | 5 | 13.389 | 5 | 0.019995 |


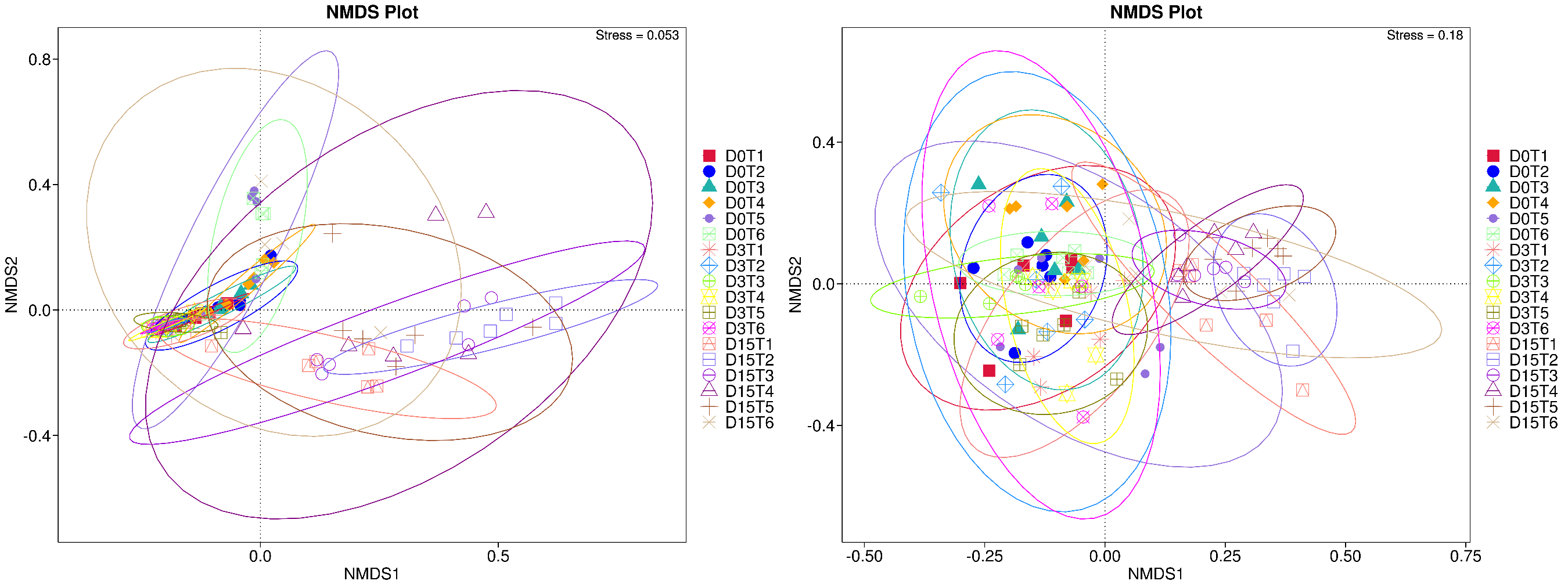


**Supplementary Figure 8** Non-Metric Multidimensional Scaling (NMDS) plots of microbial community composition. NMDS plots illustrate differences between microbial communities based on Weighted UniFrac distance at day 1 (D0T), day 7 (D3T), and day 42 (D15). 1 – low-dose, 2 – medium-dose, 3 – positive control, 4 – high-dose, 5 – enrofloxacin, 6 – negative control.

**
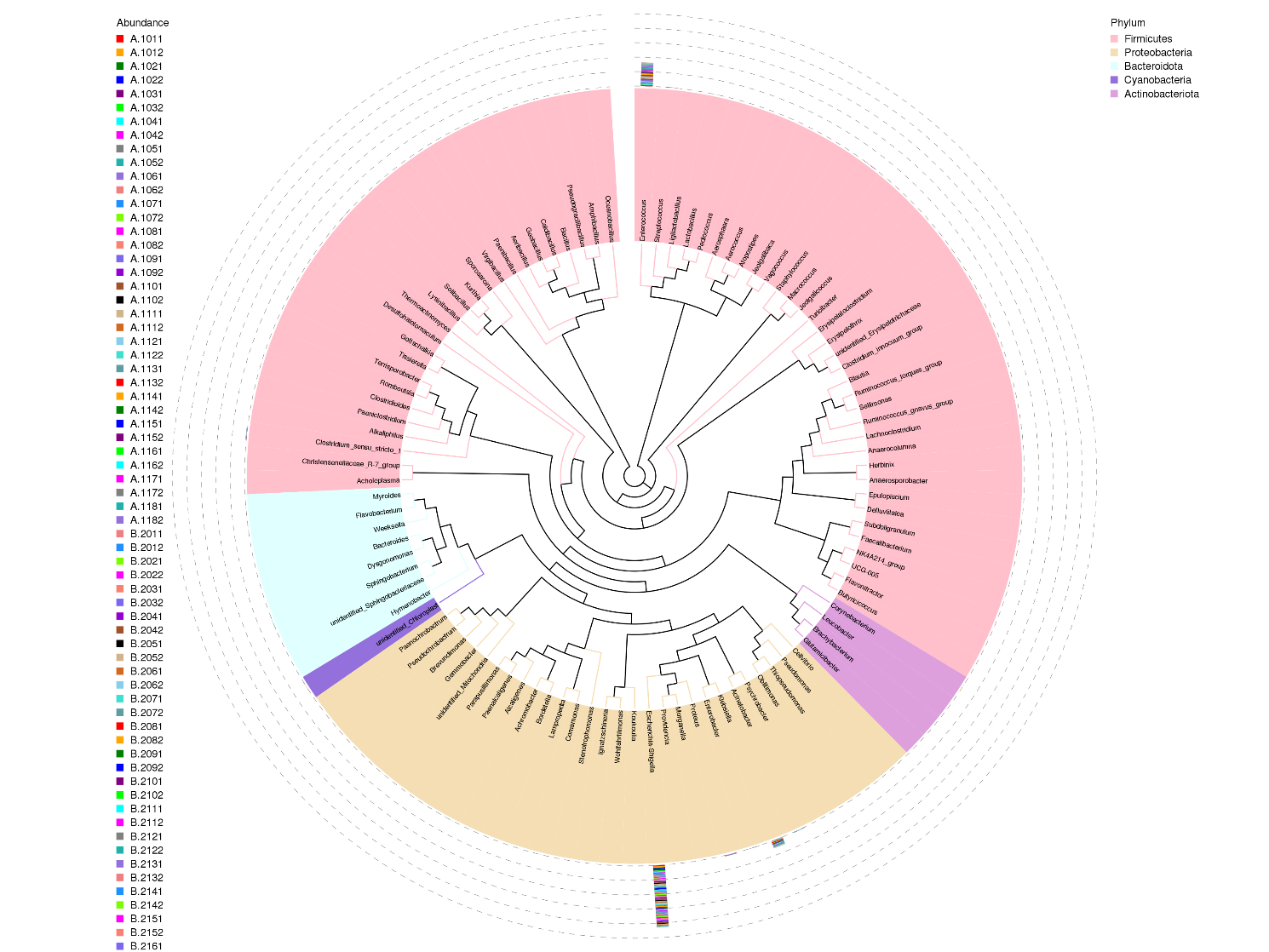
**

**Supplementary Figure 9** Phylogenetic tree of the top 100 genera. Phylogenetic relationships and relative abundances of the 100 most abundant genera are displayed. Taxa are highlighted according to their phylum-level classification.

**Supplementary Table S8.** Pairwise post hoc comparisons for alpha-diversity indices (Dunn’s test with BH–FDR correction). Pairwise differences between treatment groups (T1–T6) were assessed separately at each time point using Dunn’s post hoc test following the Kruskal–Wallis test. Reported values include group medians, Dunn’s z statistic, raw p-values, and Benjamini–Hochberg false discovery rate (BH–FDR) adjusted p-values. For readability, only comparisons reaching BH–FDR adjusted p < 0.05 are shown; the full set of pairwise comparisons is available upon request. Treatment key: T1 low-dose phytobiotic, T2 medium-dose phytobiotic, T3 positive control (infected, untreated), T4 high-dose phytobiotic, T5 enrofloxacin, T6 negative control (uninfected, untreated).

| Time point | Metric | Group 1 | Group 2 | Median (Group 1) | Median (Group 2) | Dunn z | Raw p | BH-FDR p_adj |
| --- | --- | --- | --- | --- | --- | --- | --- | --- |
| Day 1 | Dominance | T4 (high-dose) | T5 (enrofloxacin) | 0.325 | 0.5715 | -3.069 | 0.002149 | 0.03224 |
| Day 1 | Pielou's evenness | T2 (medium-dose) | T5 (enrofloxacin) | 0.4015 | 0.263 | 3 | 0.002696 | 0.034255 |
| Day 1 | Pielou's evenness | T4 (high-dose) | T5 (enrofloxacin) | 0.3835 | 0.263 | 2.836 | 0.004567 | 0.034255 |
| Day 1 | Simpson index | T4 (high-dose) | T5 (enrofloxacin) | 0.675 | 0.4285 | 3.069 | 0.002149 | 0.03224 |
| Day 7 | Dominance | T4 (high-dose) | T5 (enrofloxacin) | 0.263 | 0.781 | -4.028 | 0.000056 | 0.000845 |
| Day 7 | Dominance | T1 (low-dose) | T5 (enrofloxacin) | 0.282 | 0.781 | -3.452 | 0.000556 | 0.004168 |
| Day 7 | Dominance | T3 (positive control) | T5 (enrofloxacin) | 0.305 | 0.781 | -3.178 | 0.001481 | 0.007405 |
| Day 7 | Dominance | T2 (medium-dose) | T4 (high-dose) | 0.51 | 0.263 | 2.548 | 0.010829 | 0.040607 |
| Day 7 | Pielou's evenness | T4 (high-dose) | T5 (enrofloxacin) | 0.474 | 0.1615 | 3.686 | 0.000228 | 0.003417 |
| Day 7 | Pielou's evenness | T3 (positive control) | T5 (enrofloxacin) | 0.4735 | 0.1615 | 3.48 | 0.000501 | 0.003754 |
| Day 7 | Pielou's evenness | T1 (low-dose) | T5 (enrofloxacin) | 0.4585 | 0.1615 | 3.069 | 0.002145 | 0.010725 |
| Day 7 | Shannon diversity | T1 (low-dose) | T5 (enrofloxacin) | 2.7315 | 0.816 | 3.617 | 0.000298 | 0.002237 |
| Day 7 | Shannon diversity | T4 (high-dose) | T5 (enrofloxacin) | 2.607 | 0.816 | 3.754 | 0.000174 | 0.002237 |
| Day 7 | Shannon diversity | T3 (positive control) | T5 (enrofloxacin) | 2.4285 | 0.816 | 2.658 | 0.007866 | 0.039328 |
| Day 7 | Simpson index | T4 (high-dose) | T5 (enrofloxacin) | 0.737 | 0.219 | 4.028 | 0.000056 | 0.000845 |
| Day 7 | Simpson index | T1 (low-dose) | T5 (enrofloxacin) | 0.718 | 0.219 | 3.452 | 0.000556 | 0.004168 |
| Day 7 | Simpson index | T3 (positive control) | T5 (enrofloxacin) | 0.695 | 0.219 | 3.178 | 0.001481 | 0.007405 |
| Day 7 | Simpson index | T2 (medium-dose) | T4 (high-dose) | 0.49 | 0.737 | -2.548 | 0.010829 | 0.040607 |
| Day 42 | Chao1 richness | T5 (enrofloxacin) | T6 (negative control) | 461.1695 | 109 | 3.025 | 0.00249 | 0.037354 |
| Day 42 | Chao1 richness | T1 (low-dose) | T5 (enrofloxacin) | 280.9775 | 461.1695 | -2.733 | 0.006282 | 0.047118 |
| Day 42 | Chao1 richness | T2 (medium-dose) | T6 (negative control) | 427.0295 | 109 | 2.595 | 0.009466 | 0.04733 |
| Day 42 | Dominance | T2 (medium-dose) | T6 (negative control) | 0.0525 | 0.3 | -3.775 | 0.00016 | 0.002403 |
| Day 42 | Observed features (ASVs) | T5 (enrofloxacin) | T6 (negative control) | 452.5 | 103 | 2.987 | 0.002816 | 0.042242 |
| Day 42 | Observed features (ASVs) | T1 (low-dose) | T5 (enrofloxacin) | 273.5 | 452.5 | -2.733 | 0.006279 | 0.047091 |
| Day 42 | Pielou's evenness | T2 (medium-dose) | T6 (negative control) | 0.6575 | 0.326 | 3.809 | 0.000139 | 0.002092 |
| Day 42 | Shannon diversity | T2 (medium-dose) | T6 (negative control) | 5.644 | 2.122 | 3.949 | 0.000079 | 0.001179 |
| Day 42 | Simpson index | T2 (medium-dose) | T6 (negative control) | 0.9475 | 0.7 | 3.775 | 0.00016 | 0.002403 |

**Supplementary Table S9.** Pairwise PERMANOVA (Adonis) results based on Bray–Curtis dissimilarities. Pairwise comparisons were performed among all group–timepoint combinations (D0T1–D15T6). The table reports model F statistics, explained variance (R²), and p-values derived from permutations. P-values were adjusted across all pairwise tests using the Benjamini–Hochberg false discovery rate (BH–FDR).

| **comparison** | **df1** | **df2** | **F model** | **R2** | **p value** | **p adj bh fdr** |
| --- | --- | --- | --- | --- | --- | --- |
| D0T1-D0T2 | 1 | 10 | 1.703 | 0.146 | 0.18 | 0.189931 |
| D0T1-D0T3 | 1 | 10 | 1.116 | 0.100 | 0.301 | 0.30702 |
| D0T1-D0T4 | 1 | 10 | 7.135 | 0.416 | 0.014 | 0.0167344 |
| D0T1-D0T5 | 1 | 10 | 5.906 | 0.371 | 0.048 | 0.0544 |
| D0T1-D0T6 | 1 | 10 | 11.823 | 0.542 | 0.006 | 0.00784615 |
| D0T1-D3T1 | 1 | 10 | 11.222 | 0.529 | 0.005 | 0.00708333 |
| D0T1-D3T2 | 1 | 10 | 4.723 | 0.321 | 0.003 | 0.00651064 |
| D0T1-D3T3 | 1 | 10 | 14.811 | 0.597 | 0.002 | 0.00651064 |
| D0T1-D3T4 | 1 | 10 | 21.427 | 0.682 | 0.002 | 0.00651064 |
| D0T1-D3T5 | 1 | 10 | 18.581 | 0.650 | 0.002 | 0.00651064 |
| D0T1-D3T6 | 1 | 10 | 6.858 | 0.407 | 0.004 | 0.00651064 |
| D0T1-D15T1 | 1 | 10 | 17.738 | 0.639 | 0.004 | 0.00651064 |
| D0T1-D15T2 | 1 | 10 | 40.081 | 0.800 | 0.002 | 0.00651064 |
| D0T1-D15T3 | 1 | 10 | 12.115 | 0.548 | 0.002 | 0.00651064 |
| D0T1-D15T4 | 1 | 10 | 8.478 | 0.459 | 0.003 | 0.00651064 |
| D0T1-D15T5 | 1 | 10 | 9.659 | 0.491 | 0.005 | 0.00708333 |
| D0T1-D15T6 | 1 | 9 | 6.116 | 0.405 | 0.003 | 0.00651064 |
| D0T2-D0T3 | 1 | 10 | 0.542 | 0.051 | 0.545 | 0.552219 |
| D0T2-D0T4 | 1 | 10 | 2.192 | 0.180 | 0.128 | 0.136 |
| D0T2-D0T5 | 1 | 10 | 3.471 | 0.258 | 0.085 | 0.0928929 |
| D0T2-D0T6 | 1 | 10 | 6.365 | 0.389 | 0.028 | 0.0327023 |
| D0T2-D3T1 | 1 | 10 | 7.519 | 0.429 | 0.001 | 0.00651064 |
| D0T2-D3T2 | 1 | 10 | 5.547 | 0.357 | 0.006 | 0.00784615 |
| D0T2-D3T3 | 1 | 10 | 9.721 | 0.493 | 0.003 | 0.00651064 |
| D0T2-D3T4 | 1 | 10 | 12.487 | 0.555 | 0.003 | 0.00651064 |
| D0T2-D3T5 | 1 | 10 | 14.425 | 0.591 | 0.003 | 0.00651064 |
| D0T2-D3T6 | 1 | 10 | 6.538 | 0.395 | 0.003 | 0.00651064 |
| D0T2-D15T1 | 1 | 10 | 13.690 | 0.578 | 0.002 | 0.00651064 |
| D0T2-D15T2 | 1 | 10 | 28.901 | 0.743 | 0.003 | 0.00651064 |
| D0T2-D15T3 | 1 | 10 | 10.246 | 0.506 | 0.004 | 0.00651064 |
| D0T2-D15T4 | 1 | 10 | 7.304 | 0.422 | 0.002 | 0.00651064 |
| D0T2-D15T5 | 1 | 10 | 7.805 | 0.438 | 0.003 | 0.00651064 |
| D0T2-D15T6 | 1 | 9 | 4.688 | 0.342 | 0.006 | 0.00784615 |
| D0T3-D0T4 | 1 | 10 | 2.221 | 0.182 | 0.117 | 0.125182 |
| D0T3-D0T5 | 1 | 10 | 3.409 | 0.254 | 0.095 | 0.103085 |
| D0T3-D0T6 | 1 | 10 | 6.663 | 0.400 | 0.031 | 0.0353955 |
| D0T3-D3T1 | 1 | 10 | 8.225 | 0.451 | 0.003 | 0.00651064 |
| D0T3-D3T2 | 1 | 10 | 4.683 | 0.319 | 0.023 | 0.0270692 |
| D0T3-D3T3 | 1 | 10 | 10.211 | 0.505 | 0.002 | 0.00651064 |
| D0T3-D3T4 | 1 | 10 | 13.166 | 0.568 | 0.003 | 0.00651064 |
| D0T3-D3T5 | 1 | 10 | 13.393 | 0.573 | 0.007 | 0.008925 |
| D0T3-D3T6 | 1 | 10 | 5.398 | 0.351 | 0.005 | 0.00708333 |
| D0T3-D15T1 | 1 | 10 | 14.003 | 0.583 | 0.002 | 0.00651064 |
| D0T3-D15T2 | 1 | 10 | 30.686 | 0.754 | 0.004 | 0.00651064 |
| D0T3-D15T3 | 1 | 10 | 10.218 | 0.505 | 0.003 | 0.00651064 |
| D0T3-D15T4 | 1 | 10 | 7.383 | 0.425 | 0.005 | 0.00708333 |
| D0T3-D15T5 | 1 | 10 | 7.904 | 0.441 | 0.004 | 0.00651064 |
| D0T3-D15T6 | 1 | 9 | 5.017 | 0.358 | 0.009 | 0.0112869 |
| D0T4-D0T5 | 1 | 10 | 1.285 | 0.114 | 0.276 | 0.283409 |
| D0T4-D0T6 | 1 | 10 | 2.622 | 0.208 | 0.116 | 0.124986 |
| D0T4-D3T1 | 1 | 10 | 11.507 | 0.535 | 0.002 | 0.00651064 |
| D0T4-D3T2 | 1 | 10 | 12.708 | 0.560 | 0.003 | 0.00651064 |
| D0T4-D3T3 | 1 | 10 | 14.621 | 0.594 | 0.003 | 0.00651064 |
| D0T4-D3T4 | 1 | 10 | 17.282 | 0.633 | 0.003 | 0.00651064 |
| D0T4-D3T5 | 1 | 10 | 23.524 | 0.702 | 0.003 | 0.00651064 |
| D0T4-D3T6 | 1 | 10 | 10.409 | 0.510 | 0.005 | 0.00708333 |
| D0T4-D15T1 | 1 | 10 | 14.633 | 0.594 | 0.004 | 0.00651064 |
| D0T4-D15T2 | 1 | 10 | 30.691 | 0.754 | 0.005 | 0.00708333 |
| D0T4-D15T3 | 1 | 10 | 11.272 | 0.530 | 0.002 | 0.00651064 |
| D0T4-D15T4 | 1 | 10 | 7.754 | 0.437 | 0.002 | 0.00651064 |
| D0T4-D15T5 | 1 | 10 | 7.777 | 0.437 | 0.004 | 0.00651064 |
| D0T4-D15T6 | 1 | 9 | 4.849 | 0.350 | 0.003 | 0.00651064 |
| D0T5-D0T6 | 1 | 10 | 0.110 | 0.011 | 0.878 | 0.878 |
| D0T5-D3T1 | 1 | 10 | 8.898 | 0.471 | 0.004 | 0.00651064 |
| D0T5-D3T2 | 1 | 10 | 9.084 | 0.476 | 0.005 | 0.00708333 |
| D0T5-D3T3 | 1 | 10 | 9.669 | 0.492 | 0.003 | 0.00651064 |
| D0T5-D3T4 | 1 | 10 | 10.749 | 0.518 | 0.004 | 0.00651064 |
| D0T5-D3T5 | 1 | 10 | 10.166 | 0.504 | 0.003 | 0.00651064 |
| D0T5-D3T6 | 1 | 10 | 8.568 | 0.461 | 0.003 | 0.00651064 |
| D0T5-D15T1 | 1 | 10 | 9.682 | 0.492 | 0.003 | 0.00651064 |
| D0T5-D15T2 | 1 | 10 | 14.178 | 0.586 | 0.003 | 0.00651064 |
| D0T5-D15T3 | 1 | 10 | 7.975 | 0.444 | 0.005 | 0.00708333 |
| D0T5-D15T4 | 1 | 10 | 5.882 | 0.370 | 0.001 | 0.00651064 |
| D0T5-D15T5 | 1 | 10 | 6.496 | 0.394 | 0.006 | 0.00784615 |
| D0T5-D15T6 | 1 | 9 | 3.954 | 0.305 | 0.03 | 0.0347727 |
| D0T6-D3T1 | 1 | 10 | 14.403 | 0.590 | 0.004 | 0.00651064 |
| D0T6-D3T2 | 1 | 10 | 16.463 | 0.622 | 0.003 | 0.00651064 |
| D0T6-D3T3 | 1 | 10 | 15.988 | 0.615 | 0.004 | 0.00651064 |
| D0T6-D3T4 | 1 | 10 | 17.940 | 0.642 | 0.004 | 0.00651064 |
| D0T6-D3T5 | 1 | 10 | 21.051 | 0.678 | 0.002 | 0.00651064 |
| D0T6-D3T6 | 1 | 10 | 14.324 | 0.589 | 0.003 | 0.00651064 |
| D0T6-D15T1 | 1 | 10 | 13.706 | 0.578 | 0.003 | 0.00651064 |
| D0T6-D15T2 | 1 | 10 | 20.463 | 0.672 | 0.005 | 0.00708333 |
| D0T6-D15T3 | 1 | 10 | 11.257 | 0.530 | 0.004 | 0.00651064 |
| D0T6-D15T4 | 1 | 10 | 7.597 | 0.432 | 0.003 | 0.00651064 |
| D0T6-D15T5 | 1 | 10 | 8.636 | 0.463 | 0.004 | 0.00651064 |
| D0T6-D15T6 | 1 | 9 | 5.938 | 0.397 | 0.008 | 0.0101157 |
| D3T1-D3T2 | 1 | 10 | 6.863 | 0.407 | 0.001 | 0.00651064 |
| D3T1-D3T3 | 1 | 10 | 10.705 | 0.517 | 0.004 | 0.00651064 |
| D3T1-D3T4 | 1 | 10 | 12.927 | 0.564 | 0.001 | 0.00651064 |
| D3T1-D3T5 | 1 | 10 | 19.846 | 0.665 | 0.001 | 0.00651064 |
| D3T1-D3T6 | 1 | 10 | 3.246 | 0.245 | 0.031 | 0.0353955 |
| D3T1-D15T1 | 1 | 10 | 12.528 | 0.556 | 0.003 | 0.00651064 |
| D3T1-D15T2 | 1 | 10 | 28.147 | 0.738 | 0.004 | 0.00651064 |
| D3T1-D15T3 | 1 | 10 | 9.590 | 0.490 | 0.002 | 0.00651064 |
| D3T1-D15T4 | 1 | 10 | 7.050 | 0.413 | 0.002 | 0.00651064 |
| D3T1-D15T5 | 1 | 10 | 7.883 | 0.441 | 0.003 | 0.00651064 |
| D3T1-D15T6 | 1 | 9 | 7.171 | 0.443 | 0.001 | 0.00651064 |
| D3T2-D3T3 | 1 | 10 | 6.405 | 0.390 | 0.014 | 0.0167344 |
| D3T2-D3T4 | 1 | 10 | 9.626 | 0.490 | 0.007 | 0.008925 |
| D3T2-D3T5 | 1 | 10 | 8.346 | 0.455 | 0.002 | 0.00651064 |
| D3T2-D3T6 | 1 | 10 | 2.681 | 0.211 | 0.08 | 0.0893431 |
| D3T2-D15T1 | 1 | 10 | 12.703 | 0.560 | 0.005 | 0.00708333 |
| D3T2-D15T2 | 1 | 10 | 32.221 | 0.763 | 0.002 | 0.00651064 |
| D3T2-D15T3 | 1 | 10 | 9.797 | 0.495 | 0.001 | 0.00651064 |
| D3T2-D15T4 | 1 | 10 | 6.929 | 0.409 | 0.004 | 0.00651064 |
| D3T2-D15T5 | 1 | 10 | 8.858 | 0.470 | 0.002 | 0.00651064 |
| D3T2-D15T6 | 1 | 9 | 7.329 | 0.449 | 0.007 | 0.008925 |
| D3T3-D3T4 | 1 | 10 | 0.431 | 0.041 | 0.783 | 0.788151 |
| D3T3-D3T5 | 1 | 10 | 24.106 | 0.707 | 0.004 | 0.00651064 |
| D3T3-D3T6 | 1 | 10 | 4.490 | 0.310 | 0.011 | 0.013464 |
| D3T3-D15T1 | 1 | 10 | 8.601 | 0.462 | 0.003 | 0.00651064 |
| D3T3-D15T2 | 1 | 10 | 27.070 | 0.730 | 0.003 | 0.00651064 |
| D3T3-D15T3 | 1 | 10 | 9.209 | 0.479 | 0.004 | 0.00651064 |
| D3T3-D15T4 | 1 | 10 | 5.670 | 0.362 | 0.003 | 0.00651064 |
| D3T3-D15T5 | 1 | 10 | 8.077 | 0.447 | 0.002 | 0.00651064 |
| D3T3-D15T6 | 1 | 9 | 7.795 | 0.464 | 0.003 | 0.00651064 |
| D3T4-D3T5 | 1 | 10 | 42.826 | 0.811 | 0.002 | 0.00651064 |
| D3T4-D3T6 | 1 | 10 | 5.646 | 0.361 | 0.01 | 0.0123387 |
| D3T4-D15T1 | 1 | 10 | 9.793 | 0.495 | 0.002 | 0.00651064 |
| D3T4-D15T2 | 1 | 10 | 31.822 | 0.761 | 0.004 | 0.00651064 |
| D3T4-D15T3 | 1 | 10 | 10.332 | 0.508 | 0.003 | 0.00651064 |
| D3T4-D15T4 | 1 | 10 | 6.276 | 0.386 | 0.003 | 0.00651064 |
| D3T4-D15T5 | 1 | 10 | 8.824 | 0.469 | 0.005 | 0.00708333 |
| D3T4-D15T6 | 1 | 9 | 9.109 | 0.503 | 0.005 | 0.00708333 |
| D3T5-D3T6 | 1 | 10 | 12.839 | 0.562 | 0.002 | 0.00651064 |
| D3T5-D15T1 | 1 | 10 | 21.536 | 0.683 | 0.005 | 0.00708333 |
| D3T5-D15T2 | 1 | 10 | 46.580 | 0.823 | 0.006 | 0.00784615 |
| D3T5-D15T3 | 1 | 10 | 13.884 | 0.581 | 0.006 | 0.00784615 |
| D3T5-D15T4 | 1 | 10 | 9.262 | 0.481 | 0.003 | 0.00651064 |
| D3T5-D15T5 | 1 | 10 | 11.704 | 0.539 | 0.004 | 0.00651064 |
| D3T5-D15T6 | 1 | 9 | 7.580 | 0.457 | 0.005 | 0.00708333 |
| D3T6-D15T1 | 1 | 10 | 9.070 | 0.476 | 0.004 | 0.00651064 |
| D3T6-D15T2 | 1 | 10 | 24.840 | 0.713 | 0.002 | 0.00651064 |
| D3T6-D15T3 | 1 | 10 | 8.180 | 0.450 | 0.006 | 0.00784615 |
| D3T6-D15T4 | 1 | 10 | 5.690 | 0.363 | 0.006 | 0.00784615 |
| D3T6-D15T5 | 1 | 10 | 7.069 | 0.414 | 0.004 | 0.00651064 |
| D3T6-D15T6 | 1 | 9 | 6.665 | 0.425 | 0.004 | 0.00651064 |
| D15T1-D15T2 | 1 | 10 | 5.294 | 0.346 | 0.002 | 0.00651064 |
| D15T1-D15T3 | 1 | 10 | 1.901 | 0.160 | 0.085 | 0.0928929 |
| D15T1-D15T4 | 1 | 10 | 1.813 | 0.154 | 0.081 | 0.0898043 |
| D15T1-D15T5 | 1 | 10 | 2.137 | 0.176 | 0.016 | 0.0189767 |
| D15T1-D15T6 | 1 | 9 | 6.514 | 0.420 | 0.004 | 0.00651064 |
| D15T2-D15T3 | 1 | 10 | 2.372 | 0.192 | 0.063 | 0.070875 |
| D15T2-D15T4 | 1 | 10 | 2.663 | 0.210 | 0.006 | 0.00784615 |
| D15T2-D15T5 | 1 | 10 | 3.148 | 0.239 | 0.013 | 0.0157857 |
| D15T2-D15T6 | 1 | 9 | 11.031 | 0.551 | 0.003 | 0.00651064 |
| D15T3-D15T4 | 1 | 10 | 1.447 | 0.126 | 0.225 | 0.234184 |
| D15T3-D15T5 | 1 | 10 | 1.471 | 0.128 | 0.185 | 0.19387 |
| D15T3-D15T6 | 1 | 9 | 5.158 | 0.364 | 0.003 | 0.00651064 |
| D15T4-D15T5 | 1 | 10 | 1.390 | 0.122 | 0.248 | 0.256378 |
| D15T4-D15T6 | 1 | 9 | 3.766 | 0.295 | 0.003 | 0.00651064 |
| D15T5-D15T6 | 1 | 9 | 3.784 | 0.296 | 0.01 | 0.0123387 |

**Supplementary Table S10.** Pairwise ANOSIM results based on Bray–Curtis dissimilarities. For each pair of group–timepoint combinations, ANOSIM R statistics and permutation-based p-values are reported. BH–FDR-adjusted p-values are provided for multiple-testing control across all pairwise contrasts.

| **comparison** | **R** | **p value** | **p adj bh fdr** |
| --- | --- | --- | --- |
| D0T1-D0T2 | 0.024 | 0.317 | 0.32551 |
| D0T1-D0T3 | 0.059 | 0.241 | 0.250837 |
| D0T1-D0T4 | 0.357 | 0.023 | 0.0274922 |
| D0T1-D0T5 | 0.270 | 0.063 | 0.0703577 |
| D0T1-D0T6 | 0.520 | 0.018 | 0.022032 |
| D0T1-D3T1 | 0.869 | 0.004 | 0.00672527 |
| D0T1-D3T2 | 0.517 | 0.004 | 0.00672527 |
| D0T1-D3T3 | 0.807 | 0.002 | 0.00655714 |
| D0T1-D3T4 | 0.972 | 0.002 | 0.00655714 |
| D0T1-D3T5 | 0.943 | 0.003 | 0.00655714 |
| D0T1-D3T6 | 0.485 | 0.002 | 0.00655714 |
| D0T1-D15T1 | 0.933 | 0.002 | 0.00655714 |
| D0T1-D15T2 | 1.000 | 0.004 | 0.00672527 |
| D0T1-D15T3 | 0.637 | 0.003 | 0.00655714 |
| D0T1-D15T4 | 0.622 | 0.002 | 0.00655714 |
| D0T1-D15T5 | 0.691 | 0.004 | 0.00672527 |
| D0T1-D15T6 | 0.576 | 0.003 | 0.00655714 |
| D0T2-D0T3 | -0.048 | 0.564 | 0.567711 |
| D0T2-D0T4 | 0.128 | 0.144 | 0.154062 |
| D0T2-D0T5 | 0.191 | 0.107 | 0.116936 |
| D0T2-D0T6 | 0.354 | 0.044 | 0.0495 |
| D0T2-D3T1 | 0.706 | 0.006 | 0.00819643 |
| D0T2-D3T2 | 0.437 | 0.004 | 0.00672527 |
| D0T2-D3T3 | 0.659 | 0.002 | 0.00655714 |
| D0T2-D3T4 | 0.822 | 0.002 | 0.00655714 |
| D0T2-D3T5 | 0.770 | 0.003 | 0.00655714 |
| D0T2-D3T6 | 0.485 | 0.004 | 0.00672527 |
| D0T2-D15T1 | 0.926 | 0.004 | 0.00672527 |
| D0T2-D15T2 | 1.000 | 0.005 | 0.00735577 |
| D0T2-D15T3 | 0.648 | 0.001 | 0.00655714 |
| D0T2-D15T4 | 0.611 | 0.002 | 0.00655714 |
| D0T2-D15T5 | 0.693 | 0.004 | 0.00672527 |
| D0T2-D15T6 | 0.507 | 0.007 | 0.00907627 |
| D0T3-D0T4 | -0.033 | 0.492 | 0.50184 |
| D0T3-D0T5 | 0.224 | 0.082 | 0.090913 |
| D0T3-D0T6 | 0.378 | 0.026 | 0.0301364 |
| D0T3-D3T1 | 0.817 | 0.002 | 0.00655714 |
| D0T3-D3T2 | 0.448 | 0.019 | 0.0230714 |
| D0T3-D3T3 | 0.678 | 0.003 | 0.00655714 |
| D0T3-D3T4 | 0.880 | 0.003 | 0.00655714 |
| D0T3-D3T5 | 0.794 | 0.002 | 0.00655714 |
| D0T3-D3T6 | 0.478 | 0.003 | 0.00655714 |
| D0T3-D15T1 | 0.935 | 0.004 | 0.00672527 |
| D0T3-D15T2 | 1.000 | 0.001 | 0.00655714 |
| D0T3-D15T3 | 0.643 | 0.004 | 0.00672527 |
| D0T3-D15T4 | 0.609 | 0.005 | 0.00735577 |
| D0T3-D15T5 | 0.693 | 0.003 | 0.00655714 |
| D0T3-D15T6 | 0.533 | 0.004 | 0.00672527 |
| D0T4-D0T5 | 0.159 | 0.141 | 0.151923 |
| D0T4-D0T6 | 0.157 | 0.145 | 0.154062 |
| D0T4-D3T1 | 0.898 | 0.006 | 0.00819643 |
| D0T4-D3T2 | 0.765 | 0.002 | 0.00655714 |
| D0T4-D3T3 | 0.854 | 0.005 | 0.00735577 |
| D0T4-D3T4 | 0.926 | 0.005 | 0.00735577 |
| D0T4-D3T5 | 0.894 | 0.005 | 0.00735577 |
| D0T4-D3T6 | 0.769 | 0.003 | 0.00655714 |
| D0T4-D15T1 | 0.965 | 0.003 | 0.00655714 |
| D0T4-D15T2 | 1.000 | 0.006 | 0.00819643 |
| D0T4-D15T3 | 0.689 | 0.003 | 0.00655714 |
| D0T4-D15T4 | 0.641 | 0.001 | 0.00655714 |
| D0T4-D15T5 | 0.691 | 0.005 | 0.00735577 |
| D0T4-D15T6 | 0.571 | 0.005 | 0.00735577 |
| D0T5-D0T6 | -0.104 | 0.838 | 0.838 |
| D0T5-D3T1 | 0.650 | 0.002 | 0.00655714 |
| D0T5-D3T2 | 0.526 | 0.001 | 0.00655714 |
| D0T5-D3T3 | 0.641 | 0.002 | 0.00655714 |
| D0T5-D3T4 | 0.735 | 0.004 | 0.00672527 |
| D0T5-D3T5 | 0.628 | 0.004 | 0.00672527 |
| D0T5-D3T6 | 0.546 | 0.006 | 0.00819643 |
| D0T5-D15T1 | 0.783 | 0.003 | 0.00655714 |
| D0T5-D15T2 | 0.872 | 0.004 | 0.00672527 |
| D0T5-D15T3 | 0.683 | 0.003 | 0.00655714 |
| D0T5-D15T4 | 0.657 | 0.003 | 0.00655714 |
| D0T5-D15T5 | 0.626 | 0.004 | 0.00672527 |
| D0T5-D15T6 | 0.437 | 0.026 | 0.0301364 |
| D0T6-D3T1 | 0.793 | 0.002 | 0.00655714 |
| D0T6-D3T2 | 0.748 | 0.003 | 0.00655714 |
| D0T6-D3T3 | 0.800 | 0.003 | 0.00655714 |
| D0T6-D3T4 | 0.841 | 0.001 | 0.00655714 |
| D0T6-D3T5 | 0.811 | 0.002 | 0.00655714 |
| D0T6-D3T6 | 0.752 | 0.007 | 0.00907627 |
| D0T6-D15T1 | 0.909 | 0.003 | 0.00655714 |
| D0T6-D15T2 | 0.978 | 0.001 | 0.00655714 |
| D0T6-D15T3 | 0.804 | 0.002 | 0.00655714 |
| D0T6-D15T4 | 0.774 | 0.002 | 0.00655714 |
| D0T6-D15T5 | 0.737 | 0.007 | 0.00907627 |
| D0T6-D15T6 | 0.621 | 0.004 | 0.00672527 |
| D3T1-D3T2 | 0.663 | 0.005 | 0.00735577 |
| D3T1-D3T3 | 0.828 | 0.003 | 0.00655714 |
| D3T1-D3T4 | 0.969 | 0.002 | 0.00655714 |
| D3T1-D3T5 | 0.946 | 0.005 | 0.00735577 |
| D3T1-D3T6 | 0.319 | 0.033 | 0.0374 |
| D3T1-D15T1 | 0.917 | 0.002 | 0.00655714 |
| D3T1-D15T2 | 1.000 | 0.002 | 0.00655714 |
| D3T1-D15T3 | 0.652 | 0.007 | 0.00907627 |
| D3T1-D15T4 | 0.615 | 0.003 | 0.00655714 |
| D3T1-D15T5 | 0.672 | 0.007 | 0.00907627 |
| D3T1-D15T6 | 0.749 | 0.002 | 0.00655714 |
| D3T2-D3T3 | 0.330 | 0.032 | 0.0365373 |
| D3T2-D3T4 | 0.552 | 0.013 | 0.0160403 |
| D3T2-D3T5 | 0.581 | 0.004 | 0.00672527 |
| D3T2-D3T6 | 0.122 | 0.108 | 0.117191 |
| D3T2-D15T1 | 0.807 | 0.003 | 0.00655714 |
| D3T2-D15T2 | 1.000 | 0.005 | 0.00735577 |
| D3T2-D15T3 | 0.615 | 0.003 | 0.00655714 |
| D3T2-D15T4 | 0.556 | 0.003 | 0.00655714 |
| D3T2-D15T5 | 0.696 | 0.002 | 0.00655714 |
| D3T2-D15T6 | 0.635 | 0.006 | 0.00819643 |
| D3T3-D3T4 | -0.026 | 0.538 | 0.545126 |
| D3T3-D3T5 | 0.881 | 0.006 | 0.00819643 |
| D3T3-D3T6 | 0.333 | 0.025 | 0.0296512 |
| D3T3-D15T1 | 0.624 | 0.002 | 0.00655714 |
| D3T3-D15T2 | 1.000 | 0.003 | 0.00655714 |
| D3T3-D15T3 | 0.637 | 0.004 | 0.00672527 |
| D3T3-D15T4 | 0.483 | 0.002 | 0.00655714 |
| D3T3-D15T5 | 0.680 | 0.001 | 0.00655714 |
| D3T3-D15T6 | 0.733 | 0.005 | 0.00735577 |
| D3T4-D3T5 | 1.000 | 0.001 | 0.00655714 |
| D3T4-D3T6 | 0.537 | 0.008 | 0.0101157 |
| D3T4-D15T1 | 0.672 | 0.002 | 0.00655714 |
| D3T4-D15T2 | 1.000 | 0.003 | 0.00655714 |
| D3T4-D15T3 | 0.652 | 0.001 | 0.00655714 |
| D3T4-D15T4 | 0.496 | 0.003 | 0.00655714 |
| D3T4-D15T5 | 0.680 | 0.004 | 0.00672527 |
| D3T4-D15T6 | 0.804 | 0.003 | 0.00655714 |
| D3T5-D3T6 | 0.706 | 0.001 | 0.00655714 |
| D3T5-D15T1 | 0.961 | 0.005 | 0.00735577 |
| D3T5-D15T2 | 1.000 | 0.003 | 0.00655714 |
| D3T5-D15T3 | 0.665 | 0.004 | 0.00672527 |
| D3T5-D15T4 | 0.633 | 0.006 | 0.00819643 |
| D3T5-D15T5 | 0.746 | 0.001 | 0.00655714 |
| D3T5-D15T6 | 0.544 | 0.006 | 0.00819643 |
| D3T6-D15T1 | 0.750 | 0.001 | 0.00655714 |
| D3T6-D15T2 | 1.000 | 0.004 | 0.00672527 |
| D3T6-D15T3 | 0.593 | 0.008 | 0.0101157 |
| D3T6-D15T4 | 0.520 | 0.001 | 0.00655714 |
| D3T6-D15T5 | 0.661 | 0.005 | 0.00735577 |
| D3T6-D15T6 | 0.704 | 0.003 | 0.00655714 |
| D15T1-D15T2 | 0.624 | 0.003 | 0.00655714 |
| D15T1-D15T3 | 0.159 | 0.085 | 0.0935612 |
| D15T1-D15T4 | 0.196 | 0.023 | 0.0274922 |
| D15T1-D15T5 | 0.196 | 0.027 | 0.0310602 |
| D15T1-D15T6 | 0.800 | 0.002 | 0.00655714 |
| D15T2-D15T3 | 0.370 | 0.026 | 0.0301364 |
| D15T2-D15T4 | 0.278 | 0.007 | 0.00907627 |
| D15T2-D15T5 | 0.435 | 0.008 | 0.0101157 |
| D15T2-D15T6 | 0.909 | 0.002 | 0.00655714 |
| D15T3-D15T4 | 0.067 | 0.28 | 0.289459 |
| D15T3-D15T5 | 0.067 | 0.217 | 0.228972 |
| D15T3-D15T6 | 0.533 | 0.002 | 0.00655714 |
| D15T4-D15T5 | 0.067 | 0.237 | 0.248363 |
| D15T4-D15T6 | 0.491 | 0.009 | 0.0111951 |
| D15T5-D15T6 | 0.501 | 0.009 | 0.0111951 |

**Supplementary Table S11.** Pairwise MRPP results based on Bray–Curtis dissimilarities. The table reports the chance-corrected within-group agreement statistic (A), observed and expected delta values, and permutation-based p-values for all pairwise contrasts. BH–FDR-adjusted p-values are provided across all pairwise tests.

| **comparison** | **A** | **observed delta** | **expected delta** | **p value** | **p adj bh fdr** |
| --- | --- | --- | --- | --- | --- |
| D0T1-D0T2 | 0.020 | 0.238 | 0.24326 | 0.183 | 0.191774 |
| D0T1-D0T3 | 0.012 | 0.232 | 0.23496 | 0.283 | 0.28866 |
| D0T1-D0T4 | 0.179 | 0.234 | 0.28559 | 0.009 | 0.0111048 |
| D0T1-D0T5 | 0.201 | 0.360 | 0.45103 | 0.058 | 0.0647737 |
| D0T1-D0T6 | 0.306 | 0.304 | 0.43768 | 0.013 | 0.0155391 |
| D0T1-D3T1 | 0.283 | 0.244 | 0.34056 | 0.003 | 0.00672527 |
| D0T1-D3T2 | 0.156 | 0.213 | 0.25246 | 0.004 | 0.00672527 |
| D0T1-D3T3 | 0.333 | 0.230 | 0.34404 | 0.002 | 0.00672527 |
| D0T1-D3T4 | 0.389 | 0.208 | 0.34106 | 0.004 | 0.00672527 |
| D0T1-D3T5 | 0.387 | 0.152 | 0.24841 | 0.004 | 0.00672527 |
| D0T1-D3T6 | 0.196 | 0.249 | 0.31011 | 0.003 | 0.00672527 |
| D0T1-D15T1 | 0.382 | 0.317 | 0.51303 | 0.005 | 0.00721698 |
| D0T1-D15T2 | 0.519 | 0.282 | 0.58671 | 0.004 | 0.00672527 |
| D0T1-D15T3 | 0.326 | 0.354 | 0.52547 | 0.002 | 0.00672527 |
| D0T1-D15T4 | 0.288 | 0.427 | 0.59999 | 0.002 | 0.00672527 |
| D0T1-D15T5 | 0.308 | 0.371 | 0.53673 | 0.003 | 0.00672527 |
| D0T1-D15T6 | 0.227 | 0.354 | 0.45804 | 0.001 | 0.00672527 |
| D0T2-D0T3 | -0.021 | 0.281 | 0.27545 | 0.561 | 0.56843 |
| D0T2-D0T4 | 0.049 | 0.284 | 0.29808 | 0.119 | 0.128218 |
| D0T2-D0T5 | 0.118 | 0.409 | 0.46434 | 0.092 | 0.100543 |
| D0T2-D0T6 | 0.182 | 0.353 | 0.4316 | 0.031 | 0.0353955 |
| D0T2-D3T1 | 0.219 | 0.293 | 0.3755 | 0.004 | 0.00672527 |
| D0T2-D3T2 | 0.172 | 0.262 | 0.31651 | 0.009 | 0.0111048 |
| D0T2-D3T3 | 0.266 | 0.279 | 0.37951 | 0.008 | 0.0100328 |
| D0T2-D3T4 | 0.309 | 0.258 | 0.3726 | 0.003 | 0.00672527 |
| D0T2-D3T5 | 0.373 | 0.201 | 0.3212 | 0.003 | 0.00672527 |
| D0T2-D3T6 | 0.191 | 0.298 | 0.3688 | 0.007 | 0.00907627 |
| D0T2-D15T1 | 0.324 | 0.366 | 0.54123 | 0.004 | 0.00672527 |
| D0T2-D15T2 | 0.456 | 0.332 | 0.60944 | 0.004 | 0.00672527 |
| D0T2-D15T3 | 0.280 | 0.403 | 0.56028 | 0.004 | 0.00672527 |
| D0T2-D15T4 | 0.243 | 0.476 | 0.62903 | 0.008 | 0.0100328 |
| D0T2-D15T5 | 0.253 | 0.420 | 0.56255 | 0.006 | 0.00812389 |
| D0T2-D15T6 | 0.173 | 0.408 | 0.4931 | 0.007 | 0.00907627 |
| D0T3-D0T4 | 0.030 | 0.277 | 0.28596 | 0.2 | 0.205369 |
| D0T3-D0T5 | 0.113 | 0.403 | 0.45456 | 0.094 | 0.102 |
| D0T3-D0T6 | 0.188 | 0.347 | 0.42683 | 0.025 | 0.0289773 |
| D0T3-D3T1 | 0.230 | 0.287 | 0.37294 | 0.001 | 0.00672527 |
| D0T3-D3T2 | 0.152 | 0.256 | 0.30209 | 0.014 | 0.0166047 |
| D0T3-D3T3 | 0.268 | 0.273 | 0.37244 | 0.005 | 0.00721698 |
| D0T3-D3T4 | 0.313 | 0.251 | 0.36574 | 0.004 | 0.00672527 |
| D0T3-D3T5 | 0.354 | 0.195 | 0.30242 | 0.001 | 0.00672527 |
| D0T3-D3T6 | 0.161 | 0.292 | 0.34849 | 0.003 | 0.00672527 |
| D0T3-D15T1 | 0.324 | 0.360 | 0.53263 | 0.002 | 0.00672527 |
| D0T3-D15T2 | 0.462 | 0.325 | 0.60545 | 0.004 | 0.00672527 |
| D0T3-D15T3 | 0.278 | 0.397 | 0.54973 | 0.005 | 0.00721698 |
| D0T3-D15T4 | 0.244 | 0.470 | 0.62196 | 0.005 | 0.00721698 |
| D0T3-D15T5 | 0.254 | 0.414 | 0.55494 | 0.002 | 0.00672527 |
| D0T3-D15T6 | 0.179 | 0.401 | 0.48819 | 0.004 | 0.00672527 |
| D0T4-D0T5 | 0.053 | 0.406 | 0.42818 | 0.188 | 0.194351 |
| D0T4-D0T6 | 0.076 | 0.349 | 0.37756 | 0.134 | 0.143371 |
| D0T4-D3T1 | 0.281 | 0.289 | 0.40243 | 0.003 | 0.00672527 |
| D0T4-D3T2 | 0.303 | 0.258 | 0.37056 | 0.002 | 0.00672527 |
| D0T4-D3T3 | 0.333 | 0.275 | 0.41215 | 0.002 | 0.00672527 |
| D0T4-D3T4 | 0.358 | 0.254 | 0.3951 | 0.003 | 0.00672527 |
| D0T4-D3T5 | 0.448 | 0.198 | 0.35787 | 0.002 | 0.00672527 |
| D0T4-D3T6 | 0.266 | 0.295 | 0.40154 | 0.005 | 0.00721698 |
| D0T4-D15T1 | 0.332 | 0.362 | 0.54179 | 0.004 | 0.00672527 |
| D0T4-D15T2 | 0.461 | 0.328 | 0.60764 | 0.004 | 0.00672527 |
| D0T4-D15T3 | 0.297 | 0.399 | 0.56805 | 0.002 | 0.00672527 |
| D0T4-D15T4 | 0.253 | 0.472 | 0.63232 | 0.003 | 0.00672527 |
| D0T4-D15T5 | 0.250 | 0.417 | 0.55512 | 0.005 | 0.00721698 |
| D0T4-D15T6 | 0.177 | 0.404 | 0.49026 | 0.001 | 0.00672527 |
| D0T5-D0T6 | -0.058 | 0.475 | 0.44891 | 0.797 | 0.797 |
| D0T5-D3T1 | 0.267 | 0.415 | 0.56635 | 0.007 | 0.00907627 |
| D0T5-D3T2 | 0.266 | 0.384 | 0.52338 | 0.01 | 0.0121429 |
| D0T5-D3T3 | 0.293 | 0.401 | 0.56685 | 0.003 | 0.00672527 |
| D0T5-D3T4 | 0.322 | 0.379 | 0.55999 | 0.004 | 0.00672527 |
| D0T5-D3T5 | 0.343 | 0.323 | 0.4918 | 0.002 | 0.00672527 |
| D0T5-D3T6 | 0.247 | 0.420 | 0.5581 | 0.004 | 0.00672527 |
| D0T5-D15T1 | 0.275 | 0.488 | 0.67331 | 0.002 | 0.00672527 |
| D0T5-D15T2 | 0.355 | 0.454 | 0.70279 | 0.001 | 0.00672527 |
| D0T5-D15T3 | 0.241 | 0.525 | 0.69226 | 0.002 | 0.00672527 |
| D0T5-D15T4 | 0.195 | 0.598 | 0.74359 | 0.001 | 0.00672527 |
| D0T5-D15T5 | 0.213 | 0.542 | 0.68902 | 0.002 | 0.00672527 |
| D0T5-D15T6 | 0.149 | 0.541 | 0.63509 | 0.037 | 0.041625 |
| D0T6-D3T1 | 0.331 | 0.359 | 0.53632 | 0.002 | 0.00672527 |
| D0T6-D3T2 | 0.367 | 0.328 | 0.51769 | 0.008 | 0.0100328 |
| D0T6-D3T3 | 0.366 | 0.344 | 0.54242 | 0.002 | 0.00672527 |
| D0T6-D3T4 | 0.390 | 0.323 | 0.52919 | 0.005 | 0.00721698 |
| D0T6-D3T5 | 0.463 | 0.267 | 0.49669 | 0.004 | 0.00672527 |
| D0T6-D3T6 | 0.331 | 0.364 | 0.54362 | 0.005 | 0.00721698 |
| D0T6-D15T1 | 0.323 | 0.431 | 0.63709 | 0.007 | 0.00907627 |
| D0T6-D15T2 | 0.401 | 0.397 | 0.66232 | 0.002 | 0.00672527 |
| D0T6-D15T3 | 0.294 | 0.469 | 0.66401 | 0.004 | 0.00672527 |
| D0T6-D15T4 | 0.235 | 0.542 | 0.70861 | 0.003 | 0.00672527 |
| D0T6-D15T5 | 0.253 | 0.486 | 0.65031 | 0.005 | 0.00721698 |
| D0T6-D15T6 | 0.202 | 0.479 | 0.60062 | 0.004 | 0.00672527 |
| D3T1-D3T2 | 0.201 | 0.268 | 0.33529 | 0.003 | 0.00672527 |
| D3T1-D3T3 | 0.271 | 0.284 | 0.39031 | 0.006 | 0.00812389 |
| D3T1-D3T4 | 0.301 | 0.263 | 0.37667 | 0.003 | 0.00672527 |
| D3T1-D3T5 | 0.413 | 0.207 | 0.35297 | 0.001 | 0.00672527 |
| D3T1-D3T6 | 0.095 | 0.304 | 0.33606 | 0.021 | 0.0247154 |
| D3T1-D15T1 | 0.296 | 0.372 | 0.52781 | 0.001 | 0.00672527 |
| D3T1-D15T2 | 0.438 | 0.337 | 0.59999 | 0.002 | 0.00672527 |
| D3T1-D15T3 | 0.259 | 0.409 | 0.55216 | 0.002 | 0.00672527 |
| D3T1-D15T4 | 0.229 | 0.482 | 0.62545 | 0.002 | 0.00672527 |
| D3T1-D15T5 | 0.242 | 0.426 | 0.56216 | 0.004 | 0.00672527 |
| D3T1-D15T6 | 0.230 | 0.414 | 0.53757 | 0.005 | 0.00721698 |
| D3T2-D3T3 | 0.168 | 0.253 | 0.30446 | 0.025 | 0.0289773 |
| D3T2-D3T4 | 0.235 | 0.232 | 0.30356 | 0.01 | 0.0121429 |
| D3T2-D3T5 | 0.253 | 0.176 | 0.23589 | 0.002 | 0.00672527 |
| D3T2-D3T6 | 0.065 | 0.273 | 0.29208 | 0.084 | 0.0924604 |
| D3T2-D15T1 | 0.311 | 0.341 | 0.49432 | 0.004 | 0.00672527 |
| D3T2-D15T2 | 0.475 | 0.306 | 0.5838 | 0.007 | 0.00907627 |
| D3T2-D15T3 | 0.279 | 0.378 | 0.52379 | 0.002 | 0.00672527 |
| D3T2-D15T4 | 0.245 | 0.451 | 0.59721 | 0.004 | 0.00672527 |
| D3T2-D15T5 | 0.281 | 0.395 | 0.54914 | 0.002 | 0.00672527 |
| D3T2-D15T6 | 0.239 | 0.380 | 0.49971 | 0.003 | 0.00672527 |
| D3T3-D3T4 | -0.026 | 0.249 | 0.24251 | 0.763 | 0.76802 |
| D3T3-D3T5 | 0.453 | 0.193 | 0.35223 | 0.003 | 0.00672527 |
| D3T3-D3T6 | 0.134 | 0.290 | 0.33457 | 0.006 | 0.00812389 |
| D3T3-D15T1 | 0.238 | 0.357 | 0.46884 | 0.005 | 0.00721698 |
| D3T3-D15T2 | 0.443 | 0.323 | 0.57935 | 0.002 | 0.00672527 |
| D3T3-D15T3 | 0.266 | 0.394 | 0.53693 | 0.002 | 0.00672527 |
| D3T3-D15T4 | 0.213 | 0.468 | 0.59438 | 0.004 | 0.00672527 |
| D3T3-D15T5 | 0.260 | 0.412 | 0.55588 | 0.003 | 0.00672527 |
| D3T3-D15T6 | 0.255 | 0.398 | 0.53432 | 0.002 | 0.00672527 |
| D3T4-D3T5 | 0.538 | 0.172 | 0.37092 | 0.003 | 0.00672527 |
| D3T4-D3T6 | 0.172 | 0.269 | 0.32418 | 0.005 | 0.00721698 |
| D3T4-D15T1 | 0.261 | 0.336 | 0.45501 | 0.003 | 0.00672527 |
| D3T4-D15T2 | 0.470 | 0.302 | 0.56921 | 0.003 | 0.00672527 |
| D3T4-D15T3 | 0.293 | 0.373 | 0.52789 | 0.003 | 0.00672527 |
| D3T4-D15T4 | 0.238 | 0.446 | 0.58588 | 0.006 | 0.00812389 |
| D3T4-D15T5 | 0.280 | 0.390 | 0.54258 | 0.004 | 0.00672527 |
| D3T4-D15T6 | 0.290 | 0.375 | 0.52859 | 0.003 | 0.00672527 |
| D3T5-D3T6 | 0.336 | 0.212 | 0.31982 | 0.002 | 0.00672527 |
| D3T5-D15T1 | 0.449 | 0.280 | 0.50832 | 0.001 | 0.00672527 |
| D3T5-D15T2 | 0.570 | 0.246 | 0.57099 | 0.001 | 0.00672527 |
| D3T5-D15T3 | 0.385 | 0.317 | 0.51586 | 0.003 | 0.00672527 |
| D3T5-D15T4 | 0.334 | 0.390 | 0.58575 | 0.004 | 0.00672527 |
| D3T5-D15T5 | 0.373 | 0.334 | 0.53309 | 0.005 | 0.00721698 |
| D3T5-D15T6 | 0.276 | 0.314 | 0.43329 | 0.006 | 0.00812389 |
| D3T6-D15T1 | 0.241 | 0.377 | 0.49693 | 0.001 | 0.00672527 |
| D3T6-D15T2 | 0.419 | 0.343 | 0.58961 | 0.001 | 0.00672527 |
| D3T6-D15T3 | 0.233 | 0.414 | 0.53984 | 0.005 | 0.00721698 |
| D3T6-D15T4 | 0.199 | 0.487 | 0.60865 | 0.002 | 0.00672527 |
| D3T6-D15T5 | 0.227 | 0.431 | 0.55766 | 0.004 | 0.00672527 |
| D3T6-D15T6 | 0.215 | 0.420 | 0.53493 | 0.004 | 0.00672527 |
| D15T1-D15T2 | 0.152 | 0.410 | 0.48383 | 0.006 | 0.00812389 |
| D15T1-D15T3 | 0.048 | 0.482 | 0.50614 | 0.081 | 0.0898043 |
| D15T1-D15T4 | 0.053 | 0.555 | 0.58598 | 0.037 | 0.041625 |
| D15T1-D15T5 | 0.061 | 0.499 | 0.53138 | 0.011 | 0.013252 |
| D15T1-D15T6 | 0.201 | 0.493 | 0.61794 | 0.006 | 0.00812389 |
| D15T2-D15T3 | 0.083 | 0.447 | 0.48783 | 0.029 | 0.0333609 |
| D15T2-D15T4 | 0.088 | 0.520 | 0.57083 | 0.005 | 0.00721698 |
| D15T2-D15T5 | 0.107 | 0.465 | 0.52029 | 0.001 | 0.00672527 |
| D15T2-D15T6 | 0.296 | 0.456 | 0.64798 | 0.001 | 0.00672527 |
| D15T3-D15T4 | 0.032 | 0.592 | 0.61171 | 0.173 | 0.182545 |
| D15T3-D15T5 | 0.033 | 0.536 | 0.55442 | 0.145 | 0.154062 |
| D15T3-D15T6 | 0.166 | 0.534 | 0.64047 | 0.004 | 0.00672527 |
| D15T4-D15T5 | 0.028 | 0.609 | 0.62674 | 0.186 | 0.193592 |
| D15T4-D15T6 | 0.131 | 0.614 | 0.70623 | 0.004 | 0.00672527 |
| D15T5-D15T6 | 0.132 | 0.553 | 0.63689 | 0.008 | 0.0100328 |

**Supplementary Table S12.** Summary of log10-transformed Firmicutes-to-Bacteroidota (F/B) ratios by treatment and time point.

| Time point | Treatment code | Treatment | n (samples) | Median (IQR) log10(F/B) | Median F/B ratio* | Median Firmicutes rel. | Median Bacteroidota rel. |
| --- | --- | --- | --- | --- | --- | --- | --- |
| Day 1 | T1 | low-dose phytobiotic | 6 | 4.10 (3.82–4.31) | 13244.50 | 0.212 | 0.000 |
| Day 1 | T2 | medium-dose phytobiotic | 6 | 4.31 (4.14–4.50) | 21218.00 | 0.338 | 0.000 |
| Day 1 | T3 | positive control | 6 | 4.40 (3.83–4.58) | 25126.00 | 0.309 | 0.000 |
| Day 1 | T4 | high-dose phytobiotic | 6 | 3.92 (3.78–4.52) | 8617.25 | 0.513 | 0.000 |
| Day 1 | T5 | enrofloxacin | 6 | 3.90 (3.67–4.51) | 8089.21 | 0.735 | 0.000 |
| Day 1 | T6 | negative control | 6 | 4.73 (4.45–4.76) | 53718.00 | 0.744 | 0.000 |
| Day 42 | T1 | low-dose phytobiotic | 6 | -0.46 (-0.84–-0.26) | 0.35 | 0.066 | 0.193 |
| Day 42 | T2 | medium-dose phytobiotic | 6 | -0.42 (-0.57–-0.34) | 0.38 | 0.138 | 0.362 |
| Day 42 | T3 | positive control | 6 | -0.34 (-0.42–-0.05) | 0.46 | 0.114 | 0.221 |
| Day 42 | T4 | high-dose phytobiotic | 6 | -0.28 (-0.41–-0.04) | 0.53 | 0.103 | 0.176 |
| Day 42 | T5 | enrofloxacin | 6 | -0.26 (-0.62–0.13) | 0.61 | 0.152 | 0.274 |
| Day 42 | T6 | negative control | 5 | 3.32 (2.35–3.61) | 2096.83 | 0.644 | 0.000 |
| Day 7 | T1 | low-dose phytobiotic | 6 | 3.06 (2.40–4.07) | 1916.36 | 0.132 | 0.000 |
| Day 7 | T2 | medium-dose phytobiotic | 6 | 3.76 (3.12–3.90) | 5981.00 | 0.077 | 0.000 |
| Day 7 | T3 | positive control | 6 | 3.85 (3.76–4.00) | 7104.50 | 0.081 | 0.000 |
| Day 7 | T4 | high-dose phytobiotic | 6 | 3.77 (3.46–3.88) | 5902.00 | 0.083 | 0.000 |
| Day 7 | T5 | enrofloxacin | 6 | 3.26 (3.09–3.55) | 1847.00 | 0.114 | 0.000 |
| Day 7 | T6 | negative control | 6 | 3.08 (2.58–3.18) | 1239.00 | 0.039 | 0.000 |

**Supplementary Table S13.** Pairwise post hoc comparisons of log10(F/B) ratios using Dunn’s test with Benjamini–Hochberg false discovery rate correction within each time point.

| Time point | Treatment 1 | Treatment 2 | Comparison | z | p (Dunn) | q (BH–FDR) |
| --- | --- | --- | --- | --- | --- | --- |
| Day 1 | T1 | T2 | low-dose phytobiotic vs medium-dose phytobiotic | -0.740 | 0.4594 | 0.9085 |
| Day 1 | T1 | T3 | low-dose phytobiotic vs positive control | -0.548 | 0.5837 | 0.9085 |
| Day 1 | T1 | T4 | low-dose phytobiotic vs high-dose phytobiotic | -0.301 | 0.7631 | 0.9085 |
| Day 1 | T1 | T5 | low-dose phytobiotic vs enrofloxacin | -0.274 | 0.7841 | 0.9085 |
| Day 1 | T1 | T6 | low-dose phytobiotic vs negative control | -2.411 | 0.0159 | 0.1744 |
| Day 1 | T2 | T3 | medium-dose phytobiotic vs positive control | 0.192 | 0.8479 | 0.9085 |
| Day 1 | T2 | T4 | medium-dose phytobiotic vs high-dose phytobiotic | 0.438 | 0.6611 | 0.9085 |
| Day 1 | T2 | T5 | medium-dose phytobiotic vs enrofloxacin | 0.466 | 0.6414 | 0.9085 |
| Day 1 | T2 | T6 | medium-dose phytobiotic vs negative control | -1.671 | 0.0946 | 0.2839 |
| Day 1 | T3 | T4 | positive control vs high-dose phytobiotic | 0.247 | 0.8052 | 0.9085 |
| Day 1 | T3 | T5 | positive control vs enrofloxacin | 0.274 | 0.7841 | 0.9085 |
| Day 1 | T3 | T6 | positive control vs negative control | -1.863 | 0.0624 | 0.2341 |
| Day 1 | T4 | T5 | high-dose phytobiotic vs enrofloxacin | 0.027 | 0.9781 | 0.9781 |
| Day 1 | T4 | T6 | high-dose phytobiotic vs negative control | -2.110 | 0.0349 | 0.1744 |
| Day 1 | T5 | T6 | enrofloxacin vs negative control | -2.137 | 0.0326 | 0.1744 |
| Day 7 | T1 | T2 | low-dose phytobiotic vs medium-dose phytobiotic | -0.658 | 0.5108 | 0.6385 |
| Day 7 | T1 | T3 | low-dose phytobiotic vs positive control | -1.452 | 0.1464 | 0.4393 |
| Day 7 | T1 | T4 | low-dose phytobiotic vs high-dose phytobiotic | -0.712 | 0.4762 | 0.6385 |
| Day 7 | T1 | T5 | low-dose phytobiotic vs enrofloxacin | 0.384 | 0.7013 | 0.7514 |
| Day 7 | T1 | T6 | low-dose phytobiotic vs negative control | 0.959 | 0.3376 | 0.6329 |
| Day 7 | T2 | T3 | medium-dose phytobiotic vs positive control | -0.795 | 0.4268 | 0.6385 |
| Day 7 | T2 | T4 | medium-dose phytobiotic vs high-dose phytobiotic | -0.055 | 0.9563 | 0.9563 |
| Day 7 | T2 | T5 | medium-dose phytobiotic vs enrofloxacin | 1.041 | 0.2978 | 0.6329 |
| Day 7 | T2 | T6 | medium-dose phytobiotic vs negative control | 1.617 | 0.1060 | 0.3974 |
| Day 7 | T3 | T4 | positive control vs high-dose phytobiotic | 0.740 | 0.4594 | 0.6385 |
| Day 7 | T3 | T5 | positive control vs enrofloxacin | 1.836 | 0.0664 | 0.3974 |
| Day 7 | T3 | T6 | positive control vs negative control | 2.411 | 0.0159 | 0.2385 |
| Day 7 | T4 | T5 | high-dose phytobiotic vs enrofloxacin | 1.096 | 0.2731 | 0.6329 |
| Day 7 | T4 | T6 | high-dose phytobiotic vs negative control | 1.671 | 0.0946 | 0.3974 |
| Day 7 | T5 | T6 | enrofloxacin vs negative control | 0.575 | 0.5650 | 0.6519 |
| Day 42 | T1 | T2 | low-dose phytobiotic vs medium-dose phytobiotic | -0.113 | 0.9103 | 0.9753 |
| Day 42 | T1 | T3 | low-dose phytobiotic vs positive control | -0.901 | 0.3673 | 0.6958 |
| Day 42 | T1 | T4 | low-dose phytobiotic vs high-dose phytobiotic | -0.901 | 0.3673 | 0.6958 |
| Day 42 | T1 | T5 | low-dose phytobiotic vs enrofloxacin | -0.732 | 0.4639 | 0.6958 |
| Day 42 | T1 | T6 | low-dose phytobiotic vs negative control | -2.987 | 0.0028 | 0.0299 |
| Day 42 | T2 | T3 | medium-dose phytobiotic vs positive control | -0.789 | 0.4302 | 0.6958 |
| Day 42 | T2 | T4 | medium-dose phytobiotic vs high-dose phytobiotic | -0.789 | 0.4302 | 0.6958 |
| Day 42 | T2 | T5 | medium-dose phytobiotic vs enrofloxacin | -0.620 | 0.5354 | 0.7301 |
| Day 42 | T2 | T6 | medium-dose phytobiotic vs negative control | -2.879 | 0.0040 | 0.0299 |
| Day 42 | T3 | T4 | positive control vs high-dose phytobiotic | 0.000 | 1.0000 | 1.0000 |
| Day 42 | T3 | T5 | positive control vs enrofloxacin | 0.169 | 0.8658 | 0.9753 |
| Day 42 | T3 | T6 | positive control vs negative control | -2.127 | 0.0334 | 0.1002 |
| Day 42 | T4 | T5 | high-dose phytobiotic vs enrofloxacin | 0.169 | 0.8658 | 0.9753 |
| Day 42 | T4 | T6 | high-dose phytobiotic vs negative control | -2.127 | 0.0334 | 0.1002 |
| Day 42 | T5 | T6 | enrofloxacin vs negative control | -2.289 | 0.0221 | 0.1002 |

*F/B ratio computed as (Firmicutes + 1)/(Bacteroidota + 1) using phylum-level read counts per sample; log10(F/B) was used for statistical testing. Relative abundances are based on the proportion of reads assigned at phylum level within each sample.
